# Sulfarotene, a synthetic retinoid, overcomes stemness and sorafenib resistance of hepatocellular carcinoma via suppressing SOS2-RAS pathway

**DOI:** 10.1186/s13046-021-02085-4

**Published:** 2021-09-04

**Authors:** Feng Qi, Wenxing Qin, Yao Zhang, Yongde Luo, Bing Niu, Quanlin An, Biwei Yang, Keqing Shi, Zhijie Yu, Junwei Chen, Xin Cao, Jinglin Xia

**Affiliations:** 1 Institute of Clinical Science, Zhongshan Hospital, Fudan University, 180 Fenglin Road, 200032 Shanghai, China; 2 Liver Cancer Institute, Zhongshan Hospital, Fudan University, 180 Fenglin Road, 200032 Shanghai, China; 3 Department of Oncology, Second Affiliated Hospital of Naval Medical University, 200003 Shanghai, China; 4 Laboratory for Cellular Biomechanics and Regenerative Medicine, Department of Biomedical Engineering, College of Life Science and Technology, Huazhong University of Science and Technology, 1037 Luoyu Road, 430074 Wuhan, Hubei China; 5 The First Affiliated Hospital of Wenzhou Medical University, 325000 Wenzhou, Zhejiang China; 6 School of Pharmaceutical Sciences, Wenzhou Medical University, 325000 Wenzhou, Zhejiang China; 7 School of Life Sciences, Shanghai University, 200444 Shanghai, China

**Keywords:** Hepatocellular carcinoma, Tumor-repopulating cells, Retinoid, Sulfarotene, SOS2

## Abstract

**Background:**

Recurrent hepatocellular carcinoma (HCC) shows strong resistance to sorafenib, and the tumor-repopulating cells (TRCs) with cancer stem cell-like properties are considered a driver for its high recurrent rate and drug resistance.

**Methods:**

Suppression of TRCs may thus be an effective therapeutic strategy for treating this fatal disease. We evaluated the pharmacology and mechanism of sulfarotene, a new type of synthetic retinoid, on the cancer stem cell-like properties of HCC TRCs, and assessed its preclinical efficacy in models of HCC patient-derived xenografts (PDXs).

**Results:**

Sulfarotene selectively inhibited the growth of HCC TRCs in vitro and significantly deterred TRC-mediated tumor formation and lung metastasis in vivo without apparent toxicity, with an IC_50_ superior to that of acyclic retinoid and sorafenib, to which the recurrent HCC exhibits significant resistance at advanced stage. Sulfarotene promoted the expression and activation of RARα, which down-regulated SOS2, a key signal mediator associated with RAS activation and signal transduction involved in multiple downstream pathways. Moreover, sulfarotene selectively inhibited tumorigenesis of HCC PDXs with high expression for SOS2.

**Conclusions:**

Our study identified sulfarotene as a selective inhibitor for the TRCs of HCC, which targets a novel RARα-SOS2-RAS signal nexus, shedding light on a new, promising strategy of target therapy for advanced liver cancer.

**Supplementary Information:**

The online version contains supplementary material available at 10.1186/s13046-021-02085-4.

## Background

Hepatocellular carcinoma (HCC) is the main type of liver cancer and ranks fifth among the most common malignancies worldwide. The incidence of HCC is expected to continue rising to become the second leading cause of cancer-related deaths [1]. Although several treatment strategies for HCC are currently used in clinical practice [2], the high recurrence rate and emerging drug resistance contribute significantly to this dire situation [3].

Cancer stem cells (CSCs) or tumor-initiating cells (TICs) are a small, specialized subpopulation of cells that arise from the precancerous lesion, which play a predominant role in the progression, recurrence and resistance to therapy of HCC [4]. Instead of the complex approaches used for the identification and isolation of CSCs or TICs, e.g. by sets of stem cell surface markers [5], that hamper study applicability, we adapted and developed a simple mechanical approach to select cancer stem-like cells while they were cultured in three-dimensional (3D) soft fibrin gels [6] rather than two-dimensional (2D) rigid dishes. Such selectively survived cancer stem-like cells were shown to be highly tumorigenic, and thus were defined as tumor-repopulating cells (TRCs) [6, 7]. In a previous study, our group identified a new synthetic retinoid, named sulfarotene (WYC-209), that exerted potent, selective activity in suppressing the growth and tumor-initiating ability of TRCs derived from various types of cancer with negligible toxicity [8]. The results suggested that the combination of sulfarotene with the efficient TRC selection for cancer stem-like cells could be used to better tackle the current HCC problems as aforementioned.

In the present study, we have found that sulfarotene selectively inhibited TRCs of HCC origins and metastatic tumor formation in multiple preclinical models, including TRCs-based xenografts and patient derived xenografts (PDXs). Of note, sulfarotene effectively suppressed tumor formation and lung metastasis of the HCC TRCs that otherwise were resistance to sorafenib and ACR. Mechanistically, we demonstrated that sulfarotene upregulated RARα in HCC TRCs, which downregulated SOS2, an important mediator of oncogenic RAS activation that is critical for multiple upstream as well as downstream signaling pathways. Thus, our study has identified sulfarotene as a potential therapeutic agent for treating the TRCs of HCC by targeting a novel RARα-SOS2-RAS signal axis, which plays critical roles in tumorigenicity of TRCs and mediation of the therapeutic effects of sulfarotene.

## Materials and methods

### TRCs proliferation, migration and invasion assay

TRCs cultured in 3D fibrin gels were treated with agents, after 24 h, cell viability was assessed by using the Cell Counting Kit-8 (CCK-8, Dojindo, Japan) according to the manufacturer’s protocol in which the absorbance of produced formazan dye was measured at 450 nm. The cell inhibition rate was calculated according to the formula [(OD control cells - OD treated cells) / (OD control cells - OD blanks)] × 100. The capacity of cell migration was determined by transwell migration and invasion assay as previously described [9].

### PDX, orthotopic transplant mouse models and lung metastasis mouse models

Tumor tissues freshly isolated from patients in the operating room were dissected into small blocks of 1 mm^3^ under aseptic conditions. In a sterile environment, NOG mice were anesthetized and the HCC tissue blocks were implanted subcutaneously into the top right flank of the mice. About two months later, after reaching 1 cm in diameter, the subcutaneous PDX tumor nodes were removed, dissected into approximately 2 × 2 × 2 mm^3^ pieces, and re-transplanted into the flanks of nude mice for 30 days to permit growth as previously described [10, 11].

The mice were euthanized at day 30 or when a tumor node reached 15 mm in diameter. In metastasis experiments, the TRCs and treatment agents were injected i.v. through the tail vein into BALB/C nude mice. The lung was dissected and subjected to bioluminescence imaging in IVIS Lumina II with image radiance values normalized by the Living Image program (PerkinElmer, Boston, MA). The weight of the metastatic lung was measured and the metastasis rate was determined on paraffin embedded and HE stained lung sections by counting detectable tumor foci. PLC/PRF/5-TRCs stably overexpressing *SOS2* or shRNA targeting *SOS2* (see details in the Supplemental Materials) were established by puromycin antibiotic selection. The inoculation of these SOS2-manipulated TRCs into nude mice and subsequent drug treatment schemes essentially followed the methods described above.

### Identification of critical responsive genes by ranking and network analyses

Critical genes responsive to sulfarotene treatment were selected and ranked based on an integrated analysis of RNA-Seq and ChIP-Seq data according to the priority criteria as follows: (1) degree of neighbor nodes. The correlation of the integrated genes obtained from RNA-Seq and ChIP-Seq data (|PCCs| > 0.9 and *P* < 0.05) was determined by Pearson’s correlation coefficients (PCCs) analysis, from which a molecular network was constructed with differential degrees determined by the number of neighboring genes linked to each critical gene that was considered to play an important role in the network; (2) total number of involved pathways. After selection of a top list of associated genes according to the degree value (≥ 18), critical genes were differentially ranked based on the number of the involved biological pathways, especially those closely related to tumorigenesis, e.g., the RAS signaling pathway and pathways associated with cell cycle progression, pluripotency of stem cells and ubiquitin mediated proteolysis in the KEGG annotated pathways [12]; (3) ChIP-Seq peak values, which indicate expression values of genes in association with RARα with statistic significance (M-value ≥ 0.5, *P-*value < 0.05); (4) the membership in 3 clusters. Assuming genes downregulated in response to sulfarotene treatment as having priority roles as oncogenes, genes enriched in clusters 1, 3 and 5 based on the change patterns were considered as targets for sulfarotene treatment with a membership score ≥ 0.2.

### HCC patients recruitment and follow-up

A total of 282 patients with HCC who underwent curative resection between January 2009 and January 2010 were enrolled in 2 independent cohorts at the First Affiliated Hospital of Wenzhou Medical University (Zhejiang, China). All patients were identified by the pathologic diagnosis of HCC who had not yet received drug treatment. The clinicopathological data of all patients included the grade, stage and tumor location. However, among all the patients, 45 in cohort 1 had no follow-up while 237 were monitored until May 1, 2019, of whom 127 experienced postoperative pulmonary metastasis, as previously described [11]. Each patient provided informed consent before they participated in the study. The study protocol was approved by the Ethics Committee of The First Affiliated Hospital of Wenzhou Medical University (Zhejiang, China). The overall survival (OS) was defined as the duration from surgery to death or final follow-up, while the time to recurrence (TTR) was defined as the interval between the surgery and recurrence.

## Results

### Sulfarotene selectively targets tumor-repopulating cells of HCC *in vitro* and *in vivo*

First, sulfarotene was used to screen HCC cell lines, and effectively inhibited the proliferation of Hep3B and PLC/PRF/5 cells among the less invasive cells (Figure S1). Then, we established the TRC model with two human HCC cell lines, Hep3B and PLC/PRF/5, as previously described [6]. In view of these two HCC cell lines are weaker than other HCC cell lines in terms of proliferation and invasion, the selected TRCs from Hep3B and PLC/PRF/5 cells were more stem-like compared with their parental cancer cells. FACS analyses revealed that 89.2 and 82.1 % of the resulting Hep3B-TRCs and PLC/PRF/5-TRCs, respectively, were enriched for the expression of EpCAM^+^ and CD133^+^, two surface antigens that mark the stemness of HCC stem cells [13] (Figure S2a, b). Transwell assays revealed significantly increased survival and migration of these TRCs over their parental cancer cells *in vitro* (Figure S2c). Furthermore, all inoculums of both types of TRCs, sampled at 5 × 10^5^ cells selected from initial parental cancer cells, formed significant subcutaneous xenograft tumor nodes in BALB/c nude mice compared to none of their parental cancer cells at 30 days (Figure S2d, e), confirming the notable self-renewal and tumorigenic properties of the isolated TRCs.

**Fig. 1:**
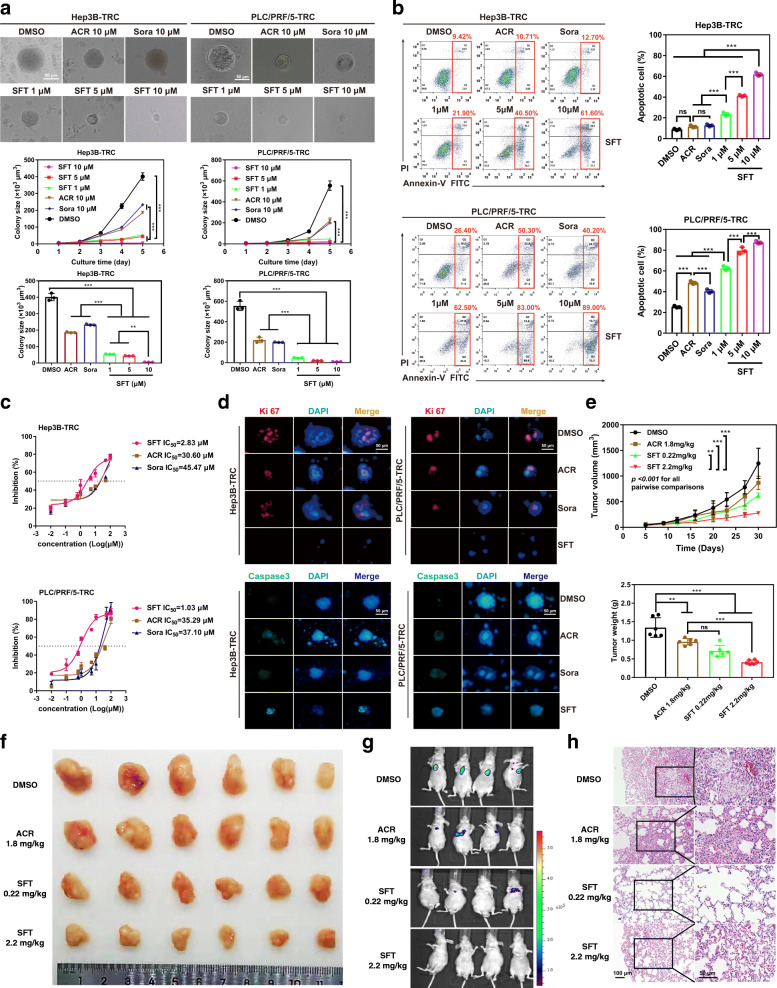
Sulfarotene selectively targets HCC TRCs.** a** Inhibition of colony spheroid formation in TRCs. Upper panel, the representative images of HCC TRC colony spheroids on day 5 (treatment day 4). Middle panel, quantitative analysis of time-dependent changes of spheroid sizes. Down panel, change of spheroid size on day 5 (*n *= 3). **b** Apoptotic effects of sulfarotene at 1.0, 5.0 and 10 µM on HCC cell derived TRCs were determined by flow-cytometry with Annexin V and propidium iodide (PI) double staining, compared with DMSO (0.1 % DMSO-containing medium), 10 µM ACR, and 10 µM sorafenib on treatment day 2 (left) (*n* = 3). Right, bar graph presentation for the percentage of apoptotic cells based on flow-cytometry analysis. **c** The IC_50_ values of sulfarotene, ACR and sorafenib for HCC TRCs were determined in the CCK8 assay after treatment for 48 h (*n* = 3). **d** Representative immunofluorescence images for the changes of Ki-67 (red) and Caspase-3 (green) in the HCC TRCs in response to treatment with 10 µM each of sulfarotene, acyclic retinoid and sorafenib for 48 h as compared to parental cancer cells. **e, f** Inhibitory effects of sulfarotene on growth and xenograft tumor node formation of Hep3B-TRCs subcutaneously transplanted to the flanks of BALB/c nude mice (*n* = 6). Five days after transplantation of TRCs, mice were treated with sulfarotene at 0.22 and 2.2 mg/kg compared to ACR at 1.8 mg/kg and the DMSO carrier. **g, h** Inhibition of lung metastasis in mice produced by sulfarotene compared to ACR. Hep3B-TRCs were injected through the tail vein into mice. After 5 days, 0.22 and 2.2 mg/kg sulfarotene and 1.8 mg/kg ACR in 0.1 % DMSO (vehicle control) in PBS were injected every 2 days for 25 days. Left, representative bioluminescence images of lung metastasis after treatment for 25 days. Right, HE staining of lung sections with TRC-derived metastasis foci (*inset*). Tukey’s post hoc test. SFT, sulfarotene; sora, sorafenib; ACR, acyclic retinoid. Data are presented as the mean ± SD; *P < 0.05, **P < 0.01, ***P < 0.001. ns, not significant

Using these HCC TRC models, we evaluated the therapeutic effects of the recently discovered RA analog sulfarotene (Figure S3) [8] in comparison with ACR, the open-ring RA analog developed almost three decades ago, sorafenib, and the solvent carrier DMSO as the negative control. After treatment for 5 days, we found that the colony spheroids from HCC TRCs were markedly suppressed by sulfarotene at concentrations ranging from 1.0 to 10 µM, compared to 10 µM for ACR or sorafenib (Fig. 1a). Similarly, sulfarotene caused a significantly higher percent of apoptotic cell deaths in both types of HCC-related TRCs compared to the other two drugs tested (Fig. 1b and Figure S4a).

Sulfarotene exhibited the lowest IC_50_ values of 2.83 and 1.08 µM of the selected Hep3B-TRCs and PLC/PRF/5-TRCs after 48 h treatment, which increased to 10.20 and 6.04 µM for Hep3B and PLC/PRF/5 cancer cells (Fig. 1c and Figure S4b). However, the IC_50_ values of sorafenib increased 2.5 and 10 fold for these two types of TRCs, respectively, which is in accordance with the reported sorafenib resistance of CSCs and in the clinic [14]. Analyses of cell proliferation and apoptosis biomarkers revealed that sulfarotene significantly reduced the expression of Ki67 while increasing Caspase-3 in TRCs compared with ACR, sorafenib and the carrier DMSO (Fig. 1d).

Next the effects of sulfarotene on the growth and formation of subcutaneous xenograft nodes derived from the inoculated Hep3B-TRCs *in vivo* in BALB/c nude mice were investigated. Both the volumes and weights of the formed Hep3B-TRCs tumor nodes were significantly reduced by sulfarotene administration every other day for a total of 25 days at a concentration of 0.22 mg/kg or 2.2 mg/kg, compared to 1.8 mg/kg for ACR and 0.1 % DMSO carrier (*P* < 0.001) (Fig. 1e, f). Five days after injection of 1 × 10^5^ Hep3B-TRCs through the tail vein, treatment with 0.22 mg/kg sulfarotene for 25 days reduced the lung metastasis foci by 50 % and 2.2 mg/kg completely blocked lung metastasis. By contrast, a dose as high as 1.8 mg/kg of ACR only reduced lung metastasis by up to 25 %, and the control of 0.1 % DMSO had no effect on lung metastasis (Fig. 1 g, h). There were no detectable toxic or necrotic effects on the heart, liver, spleen, lung or kidney tissues after sulfarotene treatment, based on structural morphology of the HE stained sections (Figure S5). Taken together, these data suggest that sulfarotene exhibits significantly better selective therapeutic activity against tumorigenesis from the HCC tumor-repopulating cells than other HCC targeted drugs.

### Sulfarotene modulates RARα to confer therapeutic sensitivity to HCC TRCs

**Fig. 2:**
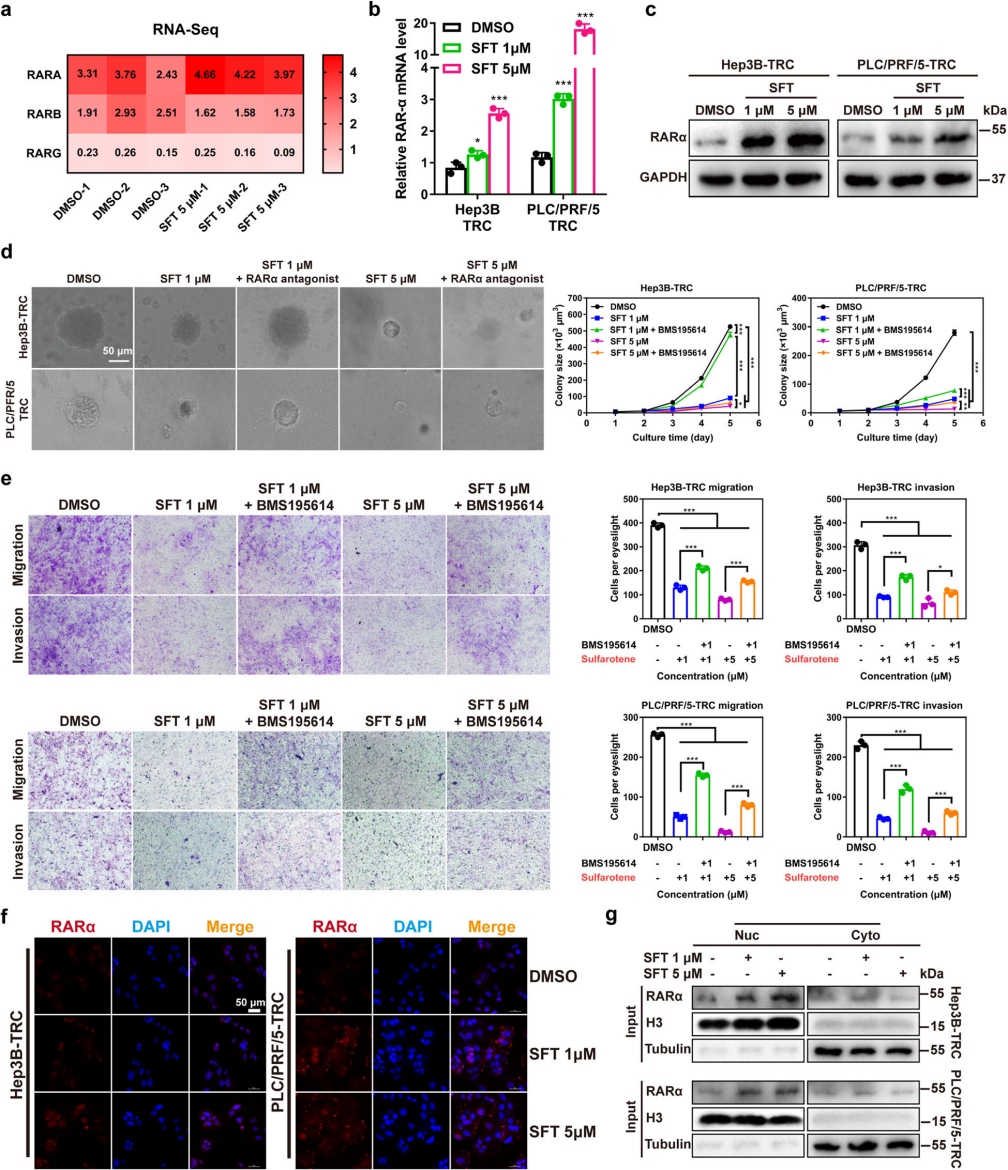
Sulfarotene potentially targets RARα in HCC TRCs.** a** RNA-Seq analysis revealed that *RARα* mRNA expression was upregulated in HCC TRCs after sulfarotene treatment compared to that of *RARβ* and *RARγ*. **b** Upregulation of *RARα* mRNA expression in HCC TRCs after sulfarotene treatment. Hep3B-TRCs and PLC/PRF/5-TRCs were treated with sulfarotene at 1.0 and 5.0 µM compared to DMSO control for 48 h. The expression levels of *RARα* relative to the control were normalized to *GAPDH* (*n* = 3). **c** Increase of RARα protein levels in Hep3B-TRCs and PLC/PRF/5-TRCs in response to dose-dependent sulfarotene treatment as revealed by western blotting. GAPDH served as the internal reference. **d** Effects of sulfarotene and an RARα antagonist on colony spheroid formation from TRCs. Tukey’s post hoc test. **e** Effects of sulfarotene and RARα antagonist on the migration and invasion abilities of TRCs. Tukey’s post hoc test. **f** Representative images of RARα (red) immunofluorescence intensity in Hep3B-TRCs and PLC/PRF/5-TRCs 48 h after treatment with sulfarotene at 1.0 and 5.0 µM. DAPI was used to counter-stain the nucleus with blue fluorescence. **g** Activation of RARα by sulfarotene as assessed by nuclear translocation. The cytosolic and nuclear fractions Hep3B-TRCs and PLC/PRF/5-TRCs after sulfarotene treatment for 48 h were isolated by ultracentrifugation. The RARα protein level in each fraction was analyzed by western blotting. Data are the mean ± SD of 3 independent experiments; *P < 0.05, **P < 0.01, ***P < 0.001

RA is an important physiological ligand of the nuclear receptors RAR and RXR that when activated exert growth inhibitory effects on various tumors [15]. However, whether sulfarotene that exerts high therapeutic selectivity and efficacy on the TRCs of HCC as described above utilizes a similar RAR- or RXR-dependent mechanism remains unclear. We first performed transcriptome sequencing and found that the mRNA levels of RARα increased under the action of sulfarotene at 5.0 µM, but not its homologs RARβ and RARγ (Fig. 2a). qPCR and western blot analyses revealed that both the mRNA and protein levels of RARα significantly increased in a dose-dependent manner in both Hep3B-TRCs and PLC/PRF/5-TRCs in response to the treatment with sulfarotene (Fig. 2b, c). Although sulfarotene treatment significantly inhibited colony formation in 3D soft fibrin gels, as well as migration and invasion in transwell culture of TRCs *in vitro*, pre-treatment with a selective RARα activation antagonist BMS195614 could significantly reverse these inhibitory effects (Fig. 2d, e). Similar results were obtained with RARα-specific siRNAs, which markedly reduced the mRNA and protein levels of RARα (Figure S6), accompanied by a reversal of the inhibitory effects imposed by sulfarotene on colony spheroid formation as well as the migration and invasion of TRCs (Figure S7a-c). As expected, immunofluorescence staining and nuclear fractionation studies revealed that sulfarotene treatment promoted the translocation of the activated RARα from the cytosol into the nucleus (Fig. 2f, g). These results strongly indicate that RARα is a potential target of sulfarotene in mediating the selective suppression of HCC TRCs.

### SOS2 potentially serves as an oncogenic factor in HCC TRCs

**Fig. 3:**
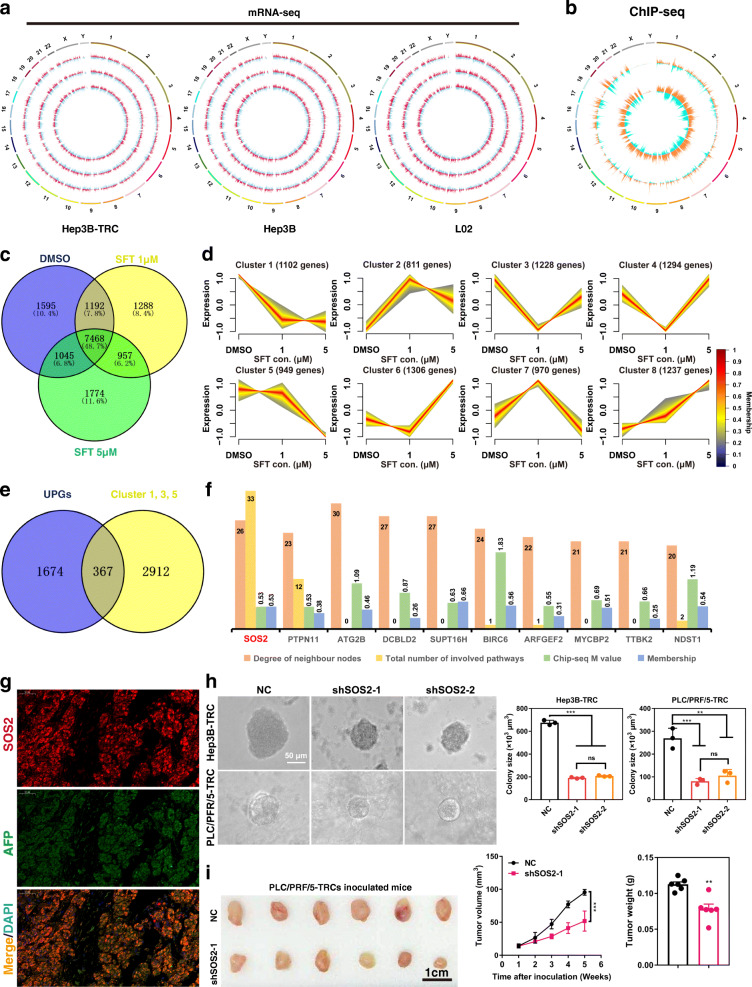
SOS2 is potentially an oncogenic factor.** a** A circular diagrams from the most outer circle to the most inner circle represent the log2 fold change value of up-regulated (red) or down-regulated (blue) differentially expressed mRNAs of Hep3B-TRC, Hep3B and L02 cells in 1 or 5 µM Sulfarotene and DMSO (0.1 % DMSO-containing medium) groups (*n* = 3, *P* < 0.05). **b** The most outer circle is the chromosome and the middle circle is the promoter that distributes the peak on the chromosome, while the most inner circle is the non-promoter that distributes the peak. The orange sample is 5 µM Sulfarotene and the blue sample is DMSO (*n* = 3, *p* < 0.05). The height represents the density of the peak at that location. **c** Venn diagrams show the number of genes in TRCs in response to treatment with 1 µM and 5 µM sulfarotene compared to 0.1 % DMSO in the medium. **d** Eight dynamic expression change patterns of union genes from the heat map in association with sulfarotene dose escalation were generated by Mfuzz. SFT con., sulfarotene concentration. **e** Venn diagrams show the number of genes identified as potential RARα-associated targets in TRCs in response to treatment with sulfarotene according to a combined analysis of RNA-Seq and ChIP-Seq data. **f** Identification of critical genes clustered in response to sulfarotene and in association with RARα. **g** Representative immunofluorescence images for the expression and co-localization of SOS2 (red) and AFP (green) among cells in the HCC tumor tissues. **h** Knockdown of *SOS2* expression by shRNAs suppressed colony spheroid growth and formation from HCC TRCs cultured in 3D fibrin gels (*n* = 3). NC, negative control shRNA. Tukey’s post hoc test. **i** Knockdown of *SOS2* expression suppressed tumor node formation derived from PLC/PRF/5-TRCs in nude mice *in vivo*, as showed by the corresponding volume and weight of the nodes (*n* = 6). Data are presented as the mean ± SD; *P < 0.05, **P < 0.01, ***P < 0.001. ns, not significant

RARα has been demonstrated to be a key transcriptional regulator of various oncogenes [16, 17]. To understand how RARα mediates the elevated therapeutic sensitivity of TRCs to sulfarotene (Fig. 1c), we analyzed the downstream target genes associated with RARα activation and sulfarotene effects by multiple integrative analyses. Multiple comparisons of clusters of the most differentially expressed genes (DEGs) were carried out with data obtained by RNA-Seq in Hep3B, Hep3B-TRCs and L02 cells treated with 0, 1.0 and 5.0 µM sulfarotene as well as by ChIP-Seq signals in Hep3B-TRCs treated with 5.0 µM sulfarotene compared to the control 0.1 % DMSO (Fig. 3a, b). The syngeneic DEGs analyses among the 3 groups classified 15,319 union genes of dynamic changes into Venn diagrams and 8 patterns (clusters 1 to 8) with Mfuzz (version 2.50.0) [18] (Fig. 3c, d).

A subsequent integrative analysis on the RNA-Seq clusters (genes from clusters 1, 3 and 5) and the ChIP-Seq signals (M-value > 0.5, *P* < 0.05) mapped 402 relevant peaks from which 367 RARα-associated genes were identified (Fig. 3e). Further ranking analyses on the 367 RARα-associated genes according to criteria with 4 priorities, including the degree of neighbor nodes, the total number of involved pathways, the ChIP-Seq peak values and the membership in clusters, identified a list of the top 10 transcriptional target genes of RARα, including *SOS2*, *PTPN11*, *ATG2B*, *DCBLD2*, *SUPT16H*, *BIRC6*, *ARFGEF2*, *MYCBP2*, *TTBK2*, and *NDST1*. Among them, *SOS2* appeared to be one of the most relevant genes in determining the highly differential sensitivity of Hep3B-TRCs to sulfarotene in association with RARα relative to Hep3B cells and L02 cells (Fig. 3f and Supplementary Data 1).

Analyses of single-cell sequencing data of 21 HCC samples obtained from the Gene Expression Omnibus (GEO, GSE149614) (Figure S8a) allowed us to identify a total of 43 clusters that could be classified into 9 cell subsets, including B cells, endothelial cells, hepatocytes, macrophage, monocyte, neurons, NK cells, smooth muscle cells and T cells (Figure S8b). However, none of these cell subsets exhibited clustered distributions with *SOS2* gene expression (Figure S8c). Consistent with the above data, we found that SOS2 protein was co-localized with tumor-specific antigen AFP in hepatoma cells but not in other cell types in human HCC tissues (Fig. 3 g).

Furthermore, analyses of the data gathered from human HCC patients by the Cancer Genome Atlas (TCGA) showed that the expression of *SOS2* in tumor foci was higher than in the paired adjacent non-tumor tissues (Figure S8d). Analyses of *SOS2* expression by qPCR from a cohort of 45 patients, as well as by IHC from a cohort of 237 HCC patients in which 127 had metastasis, revealed that the levels of SOS2 in both the primary and the metastatic tumor foci were higher than in the paired peritumor tissues (Figure S8e-h). Correlation analyses on clinical characteristics and multivariate parameters of 237 HCC patients indicated that SOS2 was an independent prognostic factor associated with both overall survival (OS) (HR = 1.442, *P* < 0.001) and time to recurrence (TTR) (HR = 1.485, *P* = 0.029) (Tables S1 and S2). Survival analyses indicated that patients who were high SOS2 expressors (comprehensive positive score (CPS) > 4) had markedly shorter median overall survival times and times to recurrence than those who were low SOS2 expressors (CPS ≤ 4) (Figure S8i, j) [19]. Functionally, reduction of SOS*2* expression by shRNAs significantly impaired the abilities of HCC TRCs to form colony spheroids and of PLC/PRF/5-TRCs to grow subcutaneous xenograft tumor nodes in nude mice (Fig. 3 h, i). Taken together, these results indicated that SOS2 is potentially oncogenic in HCC, and in particular in HCC TRCs.

### Sulfarotene overcomes stemness of HCC via suppressing SOS2

**Fig. 4:**
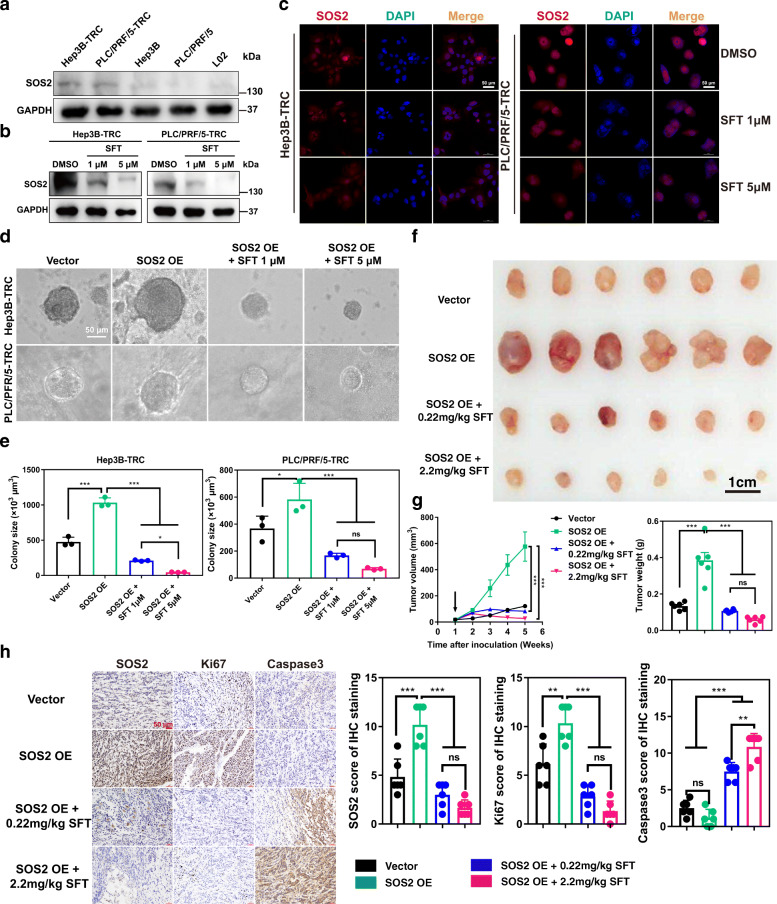
Roles of SOS2 in tumorigenesis and sensitivity to sulfarotene of HCC TRCs.** a** Higher levels of SOS2 protein were found in Hep3B-TRCs and PLC/PRF/5-TRCs than that in Hep3B, PLC/PRF/5 and L02 cells, as determined by western blotting. **b** Reduction of SOS*2* protein levels in Hep3B-TRCs and PLC/PRF/5-TRCs by treatment with sulfarotene at 1.0 and 5.0 µM for 48 h, as revealed by western blotting. GAPDH served as the loading control. **c** Suppression of SOS2 levels by sulfarotene. Representative images for immunofluorescence staining of SOS2 (red) in Hep3B-TRCs and PLC/PRF/5-TRCs cultured in 3D soft fibrin gels after treatment with sulfarotene at 1.0 and 5.0 µM for 48 h. Blue, DAPI counterstain of the nucleus. **d-e** Effects of SOS2 overexpression on colony spheroid formation and sensitivity to sulfarotene of HCC TRCs. Hep3B-TRCs and PLC/PRF/5-TRCs stably overexpressing SOS2 were treated with sulfarotene at concentrations of 1.0 and 5.0 µM. After 48 h, the size of colony spheroids was determined in µm^3^. **f-g** Effects of SOS2 overexpression on xenograft tumor node formation and sensitivity to sulfarotene of HCC TRCs. Spheroids of PLC/PRF/5-TRCs stably overexpressing SOS2 were allowed to form in 3D soft fibrin gels for 1 week, and then inoculated subcutaneously into the flanks of nude mice. A bolus injection of sulfarotene at 0.22 or 2.2 mg/kg was given i.v. to nude mice once every two days. After 4 weeks, tumor nodes (**e**) were dissected from the mice and tumor volumes and weights (**f**) were measured (*n* = 6). Down arrow indicates the start of sulfarotene treatment. **h** Effects of SOS2 overexpression on the proliferation and apoptosis of tumor nodes and sensitivity to sulfarotene of HCC TRCs. Left, representative IHC images for the expression levels of SOS2, Ki-67 and Caspase-3 in sections of xenograft tumor nodes (**e**) from PLC/PRF/5-TRCs overexpressing SOS2 or after sulfarotene treatment compared to controls. Right, statistical analysis of the IHC scores in each group (*n* = 6). Tukey’s post hoc test. Data are presented as the mean ± SD of 3 independent experiments; *P < 0.05, **P < 0.01, ***P < 0.001. ns, not significant

SOS2 (Son of Sevenless Homolog 2) is an intracellular RAS/Rho guanine nucleotide exchange factor, which promotes the exchange of RAS-GDP to RAS-GTP thereby activating RAS to allow signal transduction to multiple downstream pathways [20]. To understand the mechanistic relationship between SOS2 and the therapeutic effects of sulfarotene in association with the tumorigenic properties of HCC TRCs, we first found that the protein levels of SOS2 in TRCs were significantly higher than in their parental HCC cells and normal liver cells (Fig. 4a). Then, a significant reduction of SOS2 levels was detected in both types of TRCs in response to sulfarotene treatment in a concentration-dependent manner (Fig. 4b, c). Stable overexpression of SOS2 significantly enhanced colony spheroid formation and subsequent formation of subcutaneous xenograft tumor nodes of these TRCs. By contrast, treatment with sulfarotene at 0.22 and 2.2 mg/kg markedly repressed both effect parameters promoted by SOS2 overexpression (Fig. 4d-g). Such suppressive effects elicited by sulfarotene were associated with the loss of both SOS2 and the cell proliferation marker Ki67 and with the increase of apoptotic Caspase-3 (Fig. 4 h). These results indicated that sulfarotene potentially reduces SOS2 expression levels to elicit the observed anti-TRC and anti-tumor activity.

### Oncogenic SOS2 is transcriptionally targeted by RARα in TRCs of HCC

**Fig. 5:**
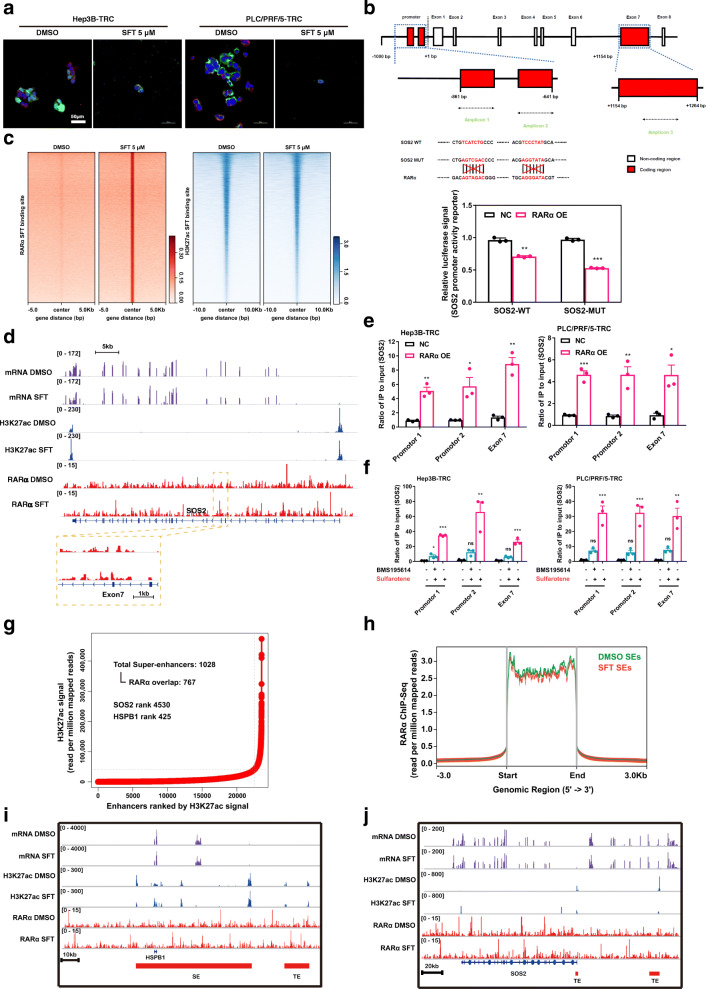
Sulfarotene inhibits SOS2 transcription via RARα.** a** Immunofluorescence showed that RARα (red) colocalized with SOS2 (green) in the nucleus of HCC TRCs treated with sulfarotene. DAPI: blue. **b** Map of the *SOS2* 5’ region from the + 1 transcription starting site. Amplicon 1 and 2, regions of interest within the SOS2 promoter in luciferase reporter experiments. Amplicons 3, region of interest in the exon 7 locus as revealed in ChIP-PCR experiments using anti-RARα antibody. **c** Heat map of RARα and H3K27ac ChIP-Seq signals in Hep3B-TRCs in response to sulfarotene treatment as compared to DMSO. Each row shows ± 5 kb centered on the RARα peak. The ChIP-Seq signal was depicted by color scaled intensity. **d** Gene tracks of ChIP-Seq signals for RNA-Seq, H3K27Ac and RARα signals around the *SOS2* loci in Hep3B-TRCs after treatment with 5.0 µM sulfarotene. ChIP-Seq and RNA-Seq signals were visualized with the Integrated Genome Viewer (IGV). **e-f** Interactions of RARα with 2 promoter elements and 1 element on exon 7 of *SOS2* gene were determined by ChIP-qPCR analysis with anti-RARα antibody and rat IgG as controls. Hep3B-TRCs and PLC/PRF/5-TRCs with stable overexpression of RARα (**e**) or treated with 5.0 µM sulfarotene in the presence or absence of BMS195614 (**f**) for 24 h were cultured for 5 more days, then ChIP-qPCR analysis was performed as described. **g** The H3K27ac signals indicated enhancers in Hep3B-TRCs treated with sulfarotene relative to DMSO. The super enhancer (SE) zone is illustrated by the dashed lines. **h** Metagene representations of RARα ChIP-Seq signals in units of read count per million mapped reads at a meta composite of SEs in Hep3B-TRCs treated with 5.0 µM sulfarotene compared to DMSO. **i-j** Gene tracks of ChIP-Seq signals for RNA-seq, H3K27Ac and RARα signals around the *HSPB1* and *SOS2* loci in Hep3B-TRCs treated with 5.0 µM sulfarotene compared to DMSO. Red bars show the sulfarotene-specific SEs and the typical enhancers (TEs)

To understand the mechanism(s) underlying the anti-tumor effect of sulfarotene through SOS2, we set about determining if RARα and *SOS2* were closely associated in response to and in mediation of sulfarotene treatment as suggested by the data shown above. Treatment with sulfarotene inhibited the expression of SOS2 while the RARα antagonist BMS195614 rescued the reduction both in Hep3B-TRCs and PLC/PRF/5-TRCs (Figure S9a). Overexpression of RARα directly lowered the expression levels of SOS2 in these TRCs (Figure S9b). Moreover, IHC analyses on the previous xenograft tumors treated with sulfarotene (Fig. 1f) revealed that SOS2 was significantly suppressed while RARα was substantially elevated upon sulfarotene treatment in comparison with ACR and the negative control DMSO (Figure S10). Nevertheless, upon sulfarotene treatment, RARα and SOS2 were observed to co-localize in the nucleuses of HCC TRCs, even though the expression of SOS2 decreased (Fig. 5a), indicating an inverse association between SOS2 and RARα.

Based on the Eukaryotic Promoter Database, analysis of the *SOS2* gene promoter region revealed 2 typical RARα binding elements located at -861 and − 641 bp, respectively (Fig. 5b), which were confirmed by a luciferase reporter assay showing that RARα overexpression decreased the luciferase activity driven by the wild-type *SOS2* promoter. Interestingly, disruption of these two elements not only failed to rescue but even further reduced the SOS2 expression driven by RARα overexpression (Fig. 5b), indicating the existence of other RARα response transcriptional regulatory region(s) in the *SOS2* gene locus. Indeed, ChIP-Seq data showed a strong increase in RARα binding due to sulfarotene treatment in Hep3B-TRCs at + 1154 to + 1264 bp of the exon 7 locus of the *SOS2* gene, while sulfarotene treatment decreased the transcriptional levels and the signals of the surrounding enhancer-like elements marked by H3K27ac of the *SOS2* gene (Fig. 5c, d). Subsequent ChIP-PCR analysis indicated that the RARα-binding sites were enriched in both the promoter and exon 7 regions on the *SOS2* gene in both TRCs (Fig. 5e, f, Figure S11 and Table S3). 3D structure simulation using the MOE program illustrated a functional binding contact between the critical amino acids (Gly-94, Gly-97, His-112, Arg-126, Leu-132, Cys-135 and Glu-160) in the DNA-binding domain of human RARα and the RARα response element in exon 7 of the *SOS2* gene (Figure S12).

Furthermore, we found that the ChIP-Seq signals for H3K27ac decreased at the *SOS2* locus in sulfarotene-treated TRCs. We speculated that the sulfarotene-induced RARα might also inhibit the super enhancers (SEs) of *SOS2* or other critical gene binding sites. By ranking according to increasing H3K27ac enrichment, we identified 1,028 SEs, among which 767 were associated with RARα (Fig. 5 g). Compared to the DMSO control, RARα binding was dramatically reduced at SEs by sulfarotene (Fig. 5 h). Although no SEs could be found at the *SOS2* gene locus, the ChIP-Seq signals for H3K27ac were found to be decreased at the typical enhancers (TEs) in the *SOS2* gene and at the SEs of *HSPB1* as a control, which has been shown to promote oncogene addiction in many types of cancer including HCC, whereas RARα increased the binding to such loci in *SOS2* and *HSPB1* in sulfarotene-treated TRCs (Fig. 5i, j). Taken together, these results suggest that SOS2 is a direct transcriptional target of RARα that is targeted by sulfarotene.

### Sulfarotene inhibits HCC TRCs by suppressing SOS2-RAS associated signaling pathways

**Fig. 6:**
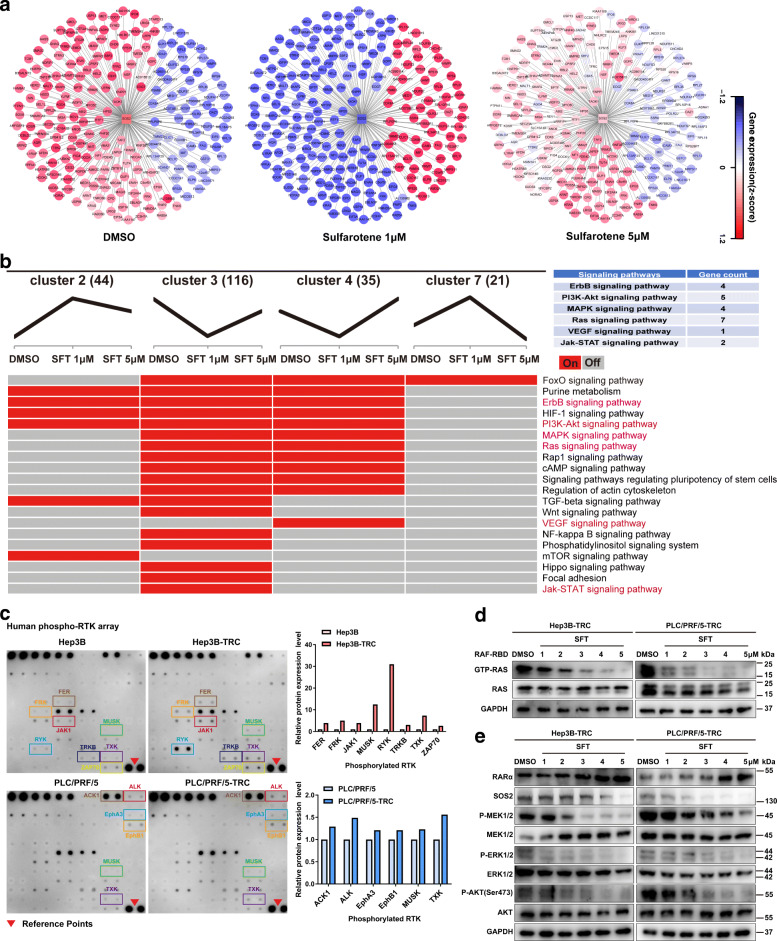
Sulfarotene targets the SOS2-RAS signal nexus.** a** Dynamic expression changes in *SOS2* and associated neighboring genes in response to sulfarotene treatment. Expression changes of *SOS2* and its 220 related genes in Hep3B-TRCs after treatment with sulfarotene at 1 and 5 µM for 2 days were revealed by RNA-Seq analysis (|PCCs| > 0.9, *P* < 0.05) and presented as a network diagram. **b** Heat map presentation of SOS2-associated KEGG pathways in response to sulfarotene treatment abstracted from 4 differential, dynamic change patterns of clusters 2, 3, 4 and 7. **c** Upregulation of phosphorylation of several types of RTKs in HCC TRCs as detected by human phospho-RTK arrays compared to that in parental HCC cancer cells. Left, representative real-time intensity images of the phospho-RTK arrays. Right, summary of the upregulated phospho-RTKs in bar graphs. **d-e** Dose-dependent inhibition of GTP-RAS, SOS2 and associated PI3K/AKT and MEK/ERK pathway mediators by sulfarotene in association with the promotion of RARα in the HCC TRCs. Hep3B-TRCs and PLC/PRF5-TRCs were treat with sulfarotene at concentrations of 1.0 to 5.0 µM for 48 h, and cell lysates were subjected to western blot analyses for the respective proteins as indicated

As SOS2 serves as a signaling center in association with RAS [20], we analyzed the possible KEGG pathways associated with SOS2-RAS activation in these HCC TRCs. Analyses of the dynamic changes of SOS2 and its 220 neighboring genes identified by RNA-Seq in response to sulfarotene treatment, according to significant Pearson correlation coefficients (PCCs), allowed us to abstract and construct a SOS2-centered participating pathway network (Fig. 6a and Supplementary Data 2). Most of the identified pathways are involved in RTK-mediated RAS signaling and downstream pathways, including PI3K/AKT and MEK/ERK (Fig. 6b). Using a human phospho-RTK array, we showed that the phosphorylation levels of several RTKs were significantly elevated compared with those of the parental cancer cells (Fig. 6c). Among the commonly associated pathway mediators downstream of these RTKs, the levels of GTP-RAS, p-MEK1/2, p-ERK1/2 and p-AKT were significantly elevated (Figure S13a, b). Sulfarotene treatment for 48 h markedly reduced the levels of both SOS2 and GTP-RAS while significantly increasing RARα, which were companied by decreases in p-MEK1/2, p-ERK1/2 and p-AKT in a concentration-dependent manner (Fig. 6d **e**). Of note, overexpression of *SOS2* significantly increased the activation of these SOS2-RAS associated pathways while sulfarotene treatment or *SOS2* knockdown effectively reversed these changes (Figure S13c-f).

### Sulfarotene overcomes sorafenib resistance via SOS2-RAS pathway suppression

**Fig. 7:**
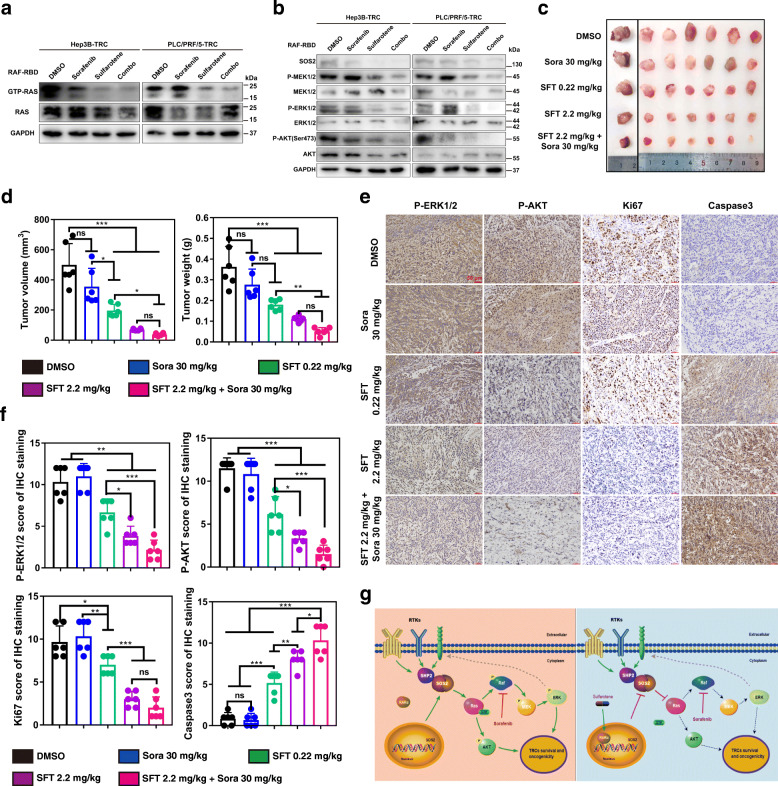
Sulfarotene inhibits *SOS2*-RAS nexus and reverses sorafenib resistance.** a** Immunoblots of whole cell lysates or RAF-RBD precipitated lysates from HCC TRCs after treatment with 5 µM sulfarotene, 10 µM sorafenib or a combination of both drugs for 48 h. **b** Effects of sulfarotene on SOS2 and RAS associated pathways compared to sorafenib. HCC TRCs were treated with sulfarotene, sorafenib or their combination for 48 h as in (**a**). Whole cell lysates were then used to assess changes in SOS2, p-MEK1/2, p-ERK1/2 and p-AKT (Ser473) relative to their corresponding total protein levels by western blotting. **c-d** Effects of sulfarotene on xenograft tumor formation of the selected HCC TRCs compared to sorafenib. 1 × 10^5^ PLC/PRF/5-TRCs were inoculated subcutaneously to the flanks in nude mice. 7 days later, 0.22 and 2.2 mg/kg sulfarotene, 30 mg/kg sorafenib or a combination of 2.2 mg/kg sulfarotene and 30 mg/kg sorafenib was injected every two days for 25 days. Sulfarotene was injected intraperitoneally while sorafenib was administered by oral intra-gastric gavage. The volume and weight (**d**) of the derived orthotopic xenograft tumor nodes (**c**) as indicated were measured. **e-f** Representative IHC images of the sections (**e**) from the orthotopic tumor nodes (**c**) indicate differential changes in the levels of p-ERK1/2, p-AKT, Ki-67 and Caspase-3 in response to treatment with 0.22 and 2.2 mg/kg sulfarotene, 30 mg/kg sorafenib, or a combination of 2.2 mg/kg sulfarotene and 30 mg/kg sorafenib. Bar graphs (**f**) show the IHC score ± SD for each group from 3 independent experiments. Tukey’s post hoc test. ***P < 0.001. ns, not significant. **g** Schema depicting the mechanism by which sulfarotene targets the RAR-SOS2-RAS signal axis to inhibit cancer cell growth and overcome drug resistance

As mentioned previously, both types of TRCs exhibited strong sorafenib resistance. We found that the phosphorylation levels of several known targets of sorafenib including PDGFRα, PDGFRβ and VEGFR3 were downregulated in PLC/PRF/5-TRCs, indicating a possible mechanism of sorafenib resistance, while the increased phosphorylation levels in the increased number of other RTKs might underlie sorafenib resistance in Hep3B-TRCs (Figure S14). Furthermore, a combination of sorafenib and sulfarotene effectively abolished the inability of sorafenib to suppress the active levels of the SOS*2*-RAS associated mediators in these HCC TRCs (Fig. 7a, b). In addition, we calculated the combination index (CI) of sulfarotene and sorafenib, and found that the two drugs have a synergistic effect (CI = 0.51 of Hep3B-TRC and CI = 0.73 of PLC/PRF/5-TRC). Further, SOS2, as the target of sulfarotene, knockdown combined with sorafenib exhibited the lower IC50 values of 7.68 µM and 14.39 µM of the selected Hep3B-TRCs and PLC/PRF/5-TRCs after 48 h treatment compared with sorafenib alone treated for HCC TRCs, which is similar to the effect of sorafenib treated for HCC cell lines (Figure S15). Consistent with the inhibition of such growth-promoting and apoptosis-suppressing pathways, the orthotopic xenograft tumors from PLC/PRF/5-TRCs inoculums succumbed to the combination regimen *in vivo* accompanied by the loss of sorafenib resistance, the decreases in p-ERK1/2 and p-AKT, and the increase in Caspase-3 (Fig. 7c-g). Collectively, the results suggested that sulfarotene effectively abolished the activation and oncogenicity of SOS2-RAS associated signaling pathways thus inhibiting the tumorigenicity and drug resistance of HCC TRCs.

### Sulfarotene inhibits tumor progression of HCC PDXs with high SOS2 expression

**Fig. 8:**
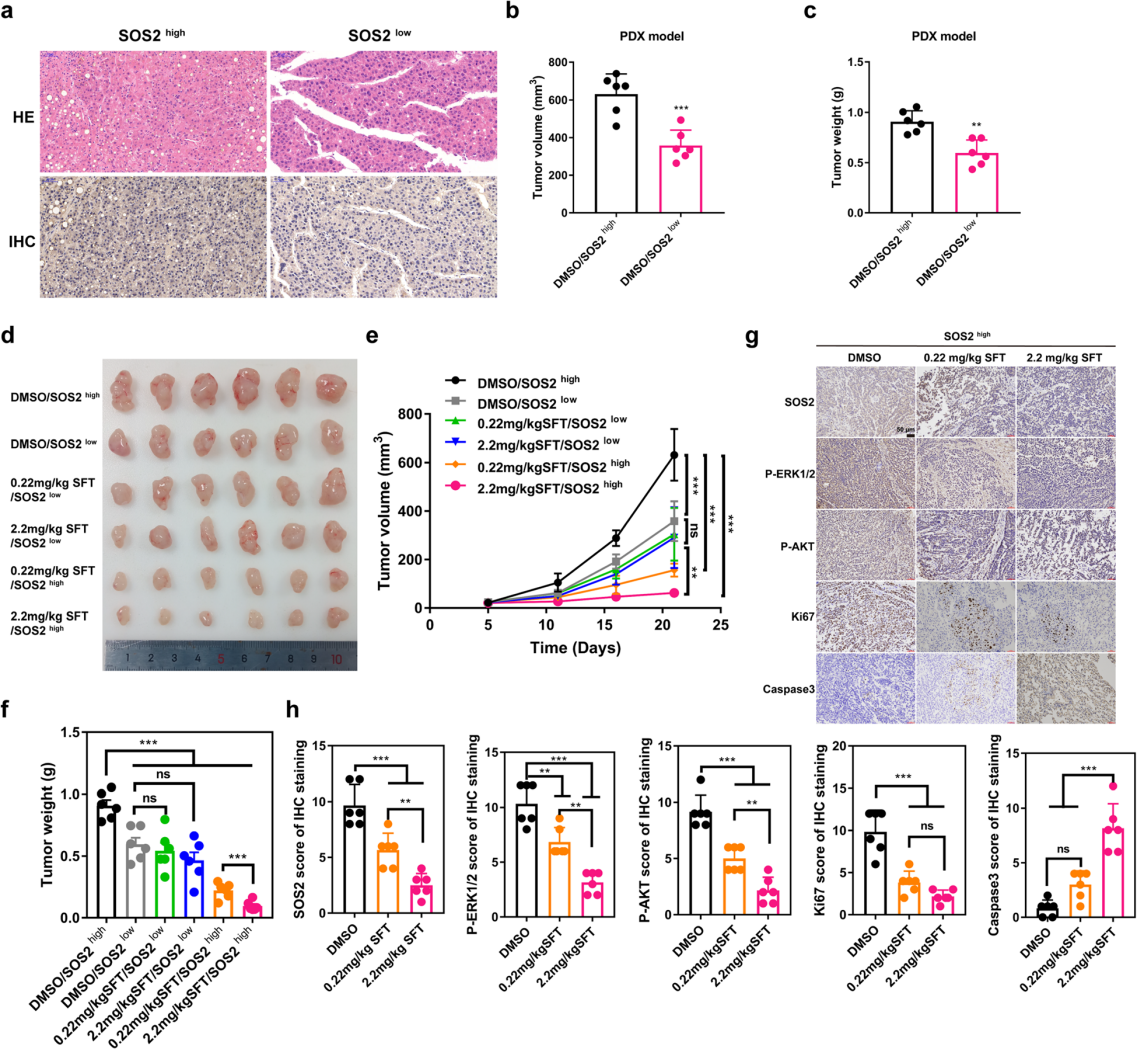
Therapeutic potential of sulfarotene on HCC PDX tumors with high expression of SOS2. **a** Representative HE images of sections of human HCC tumor foci with high and low expression levels of SOS2. **b-c** Comparative statistic analyses of the volumes and weights of tumor nodes derived from human HCC PDXs with high and low expression levels for SOS2. **d** Differential responses of human HCC PDX tumors with high and low SOS2 expression levels to sulfarotene treatment. PDX tumors from NOG mice (nonobese diabetic mice with severe combined immunodeficiency) with high (CPS > 4) or low (CPS ≤ 4) expression levels of SOS2 were reconstituted and re-transplanted subcutaneously into the flanks of athymic nude mice and subjected to treatment with sulfarotene at 0.22 and 2.2 mg/kg via i.v. injection once every two days for 3 weeks. Representative PDX-derived tumor node images are presented. **e-f** Statistic analyses of tumor volumes and weights derived from human HCC PDX tumors in (**D**) (*n* = 6). **g-h** Suppression of SOS2, RAS-associated pathways and the proliferation marker Ki67 and elevation of apoptosis marker Caspase-3 by sulfarotene treatment in HCC PDX tumors of high *SOS2* expressors. Representative IHC images of sections (**g**) and statistical analysis of IHC levels of pERK1/2, pAKT, Ki67 and Caspase-3 (**h**) in HCC PDX tumors with high expression of SOS2 (*n* = 6). Data are presented as the mean ± SD of 3 independent experiments. **P < 0.01, ***P < 0.001. ns, not significant

To determine potential therapeutic efficacy of sulfarotene on HCC, we established PDX models in NOG mice (nonobese diabetic mice with severe combined immunodeficiency) with freshly resected specimens from HCC patients. The HCC specimens were divided into one group with high expression levels (CPS > 4) and the other with low expression levels for SOS2 based on the normalized intensity of IHC stains (Fig. 8a). We found that the volume and weight of tumor nodes derived from PDXs of high SOS2 expressors were larger than that of the low SOS2 expressors, suggesting that the tumor nodes of high SOS2 expressors retained the characteristics of the HCC TRCs (Fig. 8b, c). After treatment with sulfarotene at 0, 0.22 and 2.2 mg/kg by *i.v.* injection once every 2 days for 21 days, both the volume and weight of tumor nodes of high SOS2 expressors were markedly suppressed by both the low and high dose treatments compared to that of the low SOS2 expressors and the DMSO control group (Fig. 8d-f). Sulfarotene at 2.2 mg/kg imposed a as high as 90 % reduction in the volumes of tumor nodes that originated from PDXs with high SOS2 expression compared to only a 52 % reduction in those with low SOS2 expression, demonstrating a predominant role of SOS2 in determining the malignant behavior and the high sensitivity to sulfarotene of HCC TRCs. Consistent with the aforementioned changes in key SOS2-RAS associated pathway mediators, IHC staining of tumor node sections revealed that the levels of SOS2, Ki67 and SOS2-RAS associated downstream mediators (p-MEK1/2, p-ERK1/2 and p-AKT) were remarkably inhibited by sulfarotene in the high SOS2 expressors (Fig. 8 g, h). These results suggested that sulfarotene selectively and significantly inhibited human HCC PDX tumors predominantly by blocking the SOS*2*-RAS nexus and associated signaling pathways.

## Discussion

Despite significant progress being made in the development of novel therapies, HCC remains among the most recurrent, metastatic, and thus lethal malignancies worldwide [21]. The notable self-renewal ability and high tumorigenicity of resident CSCs have been thought to be responsible for the observed high rate of recurrence, acquired drug resistance, poor prognosis, and treatment failure [6, 7, 22]. Historically, both the natural and synthetic trans-RAs have been used to treat cancers of the hemopoietic system, in particular leukemia, by targeting the nuclear receptor RAR-RXR system, however, the efficacy remains poor for solid tumors due to drug resistance, poor aqueous solubility, poor accessibility, and a short half-life [23]. Only one RA derivative, the acyclic retinoid with a prolonged half-life, has been used to treat recurrent HCV-related solid liver cancer after curative therapy by targeting oncogenic *MYCN*, which transcriptionally regulates *EPCAM*, a biomarker of HCC CSCs [24], in addition to RAR-RXR [13, 25, 26]. Much effort has led to the recent development of a new class of synthetic RA-like compound, sulfarotene (also called WYC-209), which in one study exhibited high therapeutic activity against malignant melanoma with little toxicity, which is another type of drug-resistant cancer [8]. Furthermore, a substantial portion of patients with advanced HCC also exhibits resistance to another widely used multi-kinase inhibitor sorafenib. In this endeavor, we first adapted a simple, recently developed mechanical approach to isolate CSC-like tumor-repopulating cells in 3D soft fibrin gels [6] from human HCC cell lines. We showed that the isolated TRCs recapitulated the tumorigenic and drug-resistant characteristics of CSCs while sulfarotene effectively and selectively suppressed these features *in vitro* and aborted the growth and formation of xenograft and PDX tumors and associated lung metastasis derived from TRCs inoculums *in vivo*, without notable side effects compared to ACR and sorafenib.

As a new class of structural analog of RA that serves as an agonist of RAR or RXR, sulfarotene indeed activates RARα, which is in agreement with a previous report [8]. Analyses of the dynamic, differential changes in expression of genes in response to sulfarotene treatment and of the neighboring gene network functionally associated with RARα activation, revealed SOS2 as a critical player in mediating the therapeutic effects of sulfarotene. Furthermore, we found that *SOS2* is a direct target of RARα as a transcription factor, with several typical RAR response elements to which RARα binds to repress expression. Like the family member SOS1, SOS2 belongs to the GEF family that promotes the exchange of RAS-GDP to RAS-GTP, thereby activating RAS, leading to activation of an array of downstream signaling pathways, notably the PI3K/AKT and MEK/ERK pathways, that promote cell growth, survival and migration [27]. Aberrant signaling of these pathways is associated with many types of cancers [28–30]. SOS1 was identified as a predictive gene for HCC prognosis in association with RAS [31], and a small molecular inhibitor BAY-293 that disrupts SOS1-KRAS interaction blocked RAS activation, leading to potent anti-proliferative effects [32]. Deficiency of SOS1 and SOS2 prevented malignant progression of chemical-induced skin papilloma [33]. In the present study, we found that both human HCC tumor foci and the selected TRCs were among the high expressors for SOS2, but not for SOS1, which remained low and unchanged.

Of importance, we observed that the elevated SOS2 levels or RAS activation in the selected TRCs was in accordance with increased activation of several upstream RTKs as well as downstream MEK/ERK and PI3K/AKT pathways while loss of SOS2 after sulfarotene treatment blocked the accumulation of RAS-GTP and activation of associated pathways, highlighting SOS2-RAS as a central oncogenic nexus. Apart from the aforementioned possible mechanisms, this may also explain why HCC TRCs are resistant to sorafenib while sulfarotene or a combination of sorafenib with sulfarotene effectively overcomes the resistance. Although mutations in RAS were not frequently detected in HCC, overexpression or overactivation of RTKs upstream of RAS can be prominent [34]. In addition to CSCs evolution resistance to sorafenib, there are also studies that have found that epithelial-mesenchymal transition (EMT) transition in tumor microenvironment led to sorafenib resistance. As the core factor of EMT, Snail, miR-182-5p/lncRNA-POIR and ZNF703 are highly expressed in sorafenib resistance cells, while Snail [35], miR-182-5p/lncRNA-POIR [36] and ZNF703 [37] knockdown could restore the sensitivity to sorafenib. Therefore, Regorafenib reversed sorafenib resistance by inhibiting ERK and STAT3, and subsequently downregulating Snail and EMT [38]. Moreover, dysregulated metabolism could be associated with increased sorafenib resistance in HCC [39]. Sorafenib also promoted to export glucose absorption and lactic acid [40]. PFKFB3 [41], HK2 [42], and PKM2 [43], as the key enzymes of glycolysis, have been shown to be overexpressed in sorafenib resistant HCC cell lines to increase glycolysis flux, which were silenced for contributing to synergetic effect with sorafenib. Compared to normal HCC cells, sorafenib resistant CSCs have higher rates of glucose consumption and lactate production, which are highly dependent on glycolysis [44–46]. In our study, the rates of glucose consumption and lactate production were found to be much higher in HCC TRCs, compared to the corresponding cell lines, which is consistent with other studies [47]. However, the application of sulfarotene has not resulted in the decrease of glucose consumption and lactate production in HCC TRCs, suggesting that sulfarotene could not reverse sorafenib resistance through the glycolysis pathway (Figure S16). Overall, it would be interesting to reveal the plasticity of metabolism in liver CSCs and its contribution to sorafenib resistance.

## Conclusions

Taken together, our findings have identified sulfarotene as a potentially effective agent that selectively targets HCC tumor-repopulating cells, which in many aspects resemble cancer stem cells and contribute significantly to the recurrence and drug resistance of HCC, and highlights SOS2 as a critical new oncogenic factor in association with RARα and RAS that together form a novel signal nexus in HCC.

## Supplementary Information

**Additional file 1: Supplementary Information.**

**Additional file 2: Supplementary Data 1.** The critical genes clustered in response to sulfarotene and in association with RARα.

**Additional file 3: Supplementary Data 2.** The association of SOS2 and associated neighboring genes.

## Data Availability

The obtained RNA-Seq and ChIP-Seq raw data were uploaded to the Sequence Read Archive (SRA) database of the National Center for Biotechnology Information (NCBI) (https://www.ncbi.nlm.nih.gov/), with an accession number of PRJNA673935. All data are available in the main text or in the supplementary materials.

## Abbreviations

HCC: Hepatocellular carcinoma
TRCs: Tumor-repopulating cells
PDXs: Patient-derived xenografts
CSCs: Cancer stem cells
TICs: Tumor-initiating cells
shRNA: Small hairpin RNA
OS: Overall survival
TTR: Time to recurrence
qRT-PCR: Quantitative reverse transcription-polymerase chain reaction
IHC: Immunohistochemical
