# Supplementary material for: Sulfarotene, a synthetic retinoid, overcomes stemness and sorafenib resistance of hepatocellular carcinoma via suppressing SOS2-RAS pathway
Source: J Exp Clin Cancer Res. 2021 Sep 4;40:280. doi: 10.1186/s13046-021-02085-4 (PMC8418008; doi:10.1186/s13046-021-02085-4)
Supplement: Supplementary file 1 — Additional file 1: Supplementary Information. [file 13046_2021_2085_MOESM1_ESM.docx]

**Supplementary information for the manuscript**

**Sulfarotene, a synthetic retinoid, overcomes stemness and sorafenib resistance of hepatocellular carcinoma via suppressing SOS2-RAS pathway**

Feng Qi, Wenxing Qin, Yao Zhang, Yongde Luo, Bing Niu, Quanlin An, Biwei Yang, Keqing Shi, Zhijie Yu, Junwei Chen*, Xin Cao*, Jinglin Xia*

*Correspondence: Prof. Jinglin Xia, [xiajinglin@fudan.edu.cn](mailto:xiajinglin@fudan.edu.cn); Prof. Xin Cao, [caox@fudan.edu.cn](mailto:caox@fudan.edu.cn); Prof. Junwei Chen, chenjunwei@hust.edu.cn.

**Supplementary Methods**

**Cell lines and animals**

Human liver cancer cell line Hep3B, PLC/PRF/5 and human fetal hepatocyte line L02 were purchased from the cell bank of the Chinese Academy of Sciences (Shanghai, China). Hepatoma and L02 cells were maintained in Dulbecco's Modified Eagle Medium (DMEM) (Invitrogen) with 10% fetal bovine serum (FBS) (GIBICO) at 37^o^C in a humidified incubator supplemented with 5% CO_2_.

Four-week old male nude (BALB/c *nu*/*nu*) mice were obtained from the Shanghai Institute of Material Medicine (Shanghai, China), Chinese Academy of Science. All mice were randomly allocated to a control group or treatment group (n = 6). The animal study protocols were performed in accordance with the Guide for the Care and Use of Laboratory Animals stipulated by the National Academy of Sciences and the National Institutes of Health (NIH publication 86-23, revised 1985) and approved by the Animal Care and Use Committee of Zhongshan Hospital, Fudan University, Shanghai, China.

**Selection and culture of TRCs *in* *vitro***

3D soft fibrin gels with a stiffness of 90 Pa were prepared with salmon fibrinogen and thrombin as previously described (1-3). Briefly, HCC cell lines, including Hep3B and PLC/PRF/5, were cultured on conventional 2D rigid plates until 80-90% confluence and then were detached with 0.25% trypsin. A single cell suspension of 1 × 10^4^ cells/mL was mixed with an equal volume of 2 mg/mL fibrinogen solution diluted with T7 buffer (50 mM Tris-HCl, 150 mM NaCl, pH 7.4). 5 μL thrombin (100 U/mL) was added to each well containing a 250 μL mixture of fibrinogen and cells in a 24-well plate. The cell plate was placed in 37^o^C cell culture incubator for 50 min, followed by the addition of 1 ml DMEM medium containing 10% FBS. After 24 h, the formation of TRC colonies was continuously monitored daily under an inverted microscope.

**Reagents**

Sulfarotene was prepared in our laboratory as previously described (*13*). ACR (peretinoin, HY-100008) and sorafenib (HY-10201) were purchased from MedChemExpress. RARα antibody (Ab) (sc-515796) was purchased from Santa Cruz, SOS2 (AF5237) from Affinity and RAS Ab (ab16907) and GAPDH Ab (ab8245) were sourced from Abcam. Antibodies for MEK1/2 (4694), p-MEK1/2 (9121), ERK1/2 (4695), p-ERK (4370S), AKT (2920), p-AKT (ser473), Histone H3 (4499), α-Tubulin (2125), Ki67 (9449) and cleaved Caspase-3 (9661) were sourced from Cell Signaling Technology. FITC anti-human CD326 (EpCAM) Ab (324204) was purchased from Biolengend. HRP-conjugated secondary antibodies were sourced from Jackson ImmunoResearch Laboratories (West Grove, PA, USA).

**Flow cytometry analysis**

After treatment, TRCs cells were centrifuged at 1,000 rpm for 5 min to remove the culture medium. Cell pellets were washed twice with PBS, incubated with 5 μL FITC anti-human CD326 or FITC-annexin V and PI (Propidium Iodide) for 30 min at 4^o^C, and then subject to flow cytometry analysis according to the manufacturer’s instructions (Sigma, USA). For cell cycle analysis, after washing with PBS, TRCs were subject to fixation in ice-cold 70% ethanol for 30 min, resuspended in PBS containing RNA enzyme (0.25 mg/mL), incubated with 5 μL PI at room temperature for 30 min, and then analyzed by flow cytometry (Calibur, BD biosciences) with FlowJo software.

**Quantitative PCR**

Total RNAs were isolated from HCC tissues and TRCs using Trizol (Takara, Japan), followed by reverse transcription for 1st strand cDNA synthesis with PrimeScript RT reagent kit (Takara, Japan) according to the manufacturer's instructions. Expression changes of genes were measured using SYBR^®^ Premix ExTaq™ (Takara, Japan) with a reaction condition of initial denaturation at 95^o^C for 5 min and then 40 cycles of

95^o^C for 10 sec and 60^o^C for 30 sec on an ABI Prism 7500 sequence detection system (Applied Biosystems, Foster City, CA, USA). The comparative threshold 2^−ΔΔCT^ method was used with GAPDH as the internal reference. The gene primers (Servicebio technology Co., LTD, Wuhan, China) used were as follows:

*GAPDH* forward primer 5‘-GGAAGCTTGTCATCAATGGAAATC-3‘ and reverse primer 5’- TGATGACCCTTTTGGCTCCC-3’, *RARα* forward primer 5‘-CCCTCTACCCCGCATCTACA-3‘ and reverse primer 5’-CTTCTTGTTTCGGTCGTTTCTC-3’, *SOS2* forward primer 5‘- AGGCAAACGGAGTAAGCCATAA-3‘ and reverse primer 5’- GGTGAGATTTGTGGTATGGCG-3’, *EPCAM* forward primer 5‘- GTGCTGGTGTGTGAACACTG-3‘ and reverse primer 5’- TCTGAAGTGCAGTCCGCAA-3’, *CD133* forward primer 5‘- GTACAACGCCAAACCACGACT-3‘ and reverse primer 5’- CGCACACGCCACACAGTAA-3’, *Nanog* forward primer 5‘- AATGGTGTGACGCAGGGATG-3‘ and reverse primer 5’- TGCACCAGGTCTGAGTGTTC-3’, *Sox2* forward primer 5‘- CCTACAGCATGTCCTACTCGCA-3‘ and reverse primer 5’- CTGGAGTGGGAGGAAGAGGTAAC-3’, *CD44* forward primer 5‘- GAAAGGAGCAGCACTTCAGGA-3‘ and reverse primer 5’- TTCTTGCCTCTTGGTTGCTGT-3’, and *CD90* forward primer 5‘- CGCTCTCCTGCTAACAGTCTTG-3‘ and reverse primer 5’- TGGATGGGTGAACTGCTGGTAT-3’.

**Western blotting**

Tissues and cells were homogenized in RIPA extraction buffer (Pierce Biotechnology, Rockford, IL, USA) and the protein extracts were separated on 10% SDS-PAGE and then transferred onto polyvinylidene difluoride (PVDF) membranes, which were then blocked with 5% non-fat milk for 1 h. The membranes were then probed sequentially with primary antibodies and HRP-conjugated secondary antibodies, and protein bands were detected by application of ECL reagent (Tiangen, Beijing, China) and visualized by autoradiography in an Odyssey Imaging System (LiCor Biosciences, Lincoln, NE). ImageJ densitometry program was used to quantify individual bands.

**Immunofluorescence**

TRCs were incubated in the confocal dish, fixed by 4% paraformaldehyde, and then permeated by treating with 0.5% Triton X-100 in PBS at room temperature for 20 min. The primary antibodies were replenished in the dish overnight at 4^o^C, and then secondary antibodies were added to the incubation, which continued at room temperature for 50 min. The cells on the culture slides were then analyzed under a fluorescence microscope (Olympus, Tokyo, Japan).

**RNA interference and lentivirus-mediated cell transduction**

Cells were transfected with siRNA using riboFECT™ CP ([C10511-05](https://www.ribobio.com/product_detail/?sku=C10511-05), RIBO biological corporation, Guangdong, China) according to the manufacturer’s protocol. The non-target siRNA control ([siN0000001-1-10](https://www.ribobio.com/product_detail/?sku=siN0000001-1-10), RIBO biological corporation, Guangdong, China) was used as a negative control. Sequences of the siRNAs directed against RARα include stB0005373A (GTGAGAAACGACCGAAACA), stB0005373B (CTCAGAACAACGTGTCTCT), and stB0005373C (ATTACTGACCTGCGAAGCA).

Plasmids for stable overexpression and knockdown of SOS2 were established using a lentiviral-mediated expression system, which was purchased from GeneChem corporation (Shanghai, China). Sequences of the three shRNAs directed against SOS2 include LV-shRNA-1 (GGTACAAAGTGGACTACCA), LV-shRNA-2 (GGACGAAGCTGTGGAATTA), and LV-shRNA-3 (CAAGGAATATGTCCAACCA). The efficiency of cell transfection was examined by western blot analysis.

**Immunohistochemistry and tissue microarrays analysis**

Immunohistochemical staining (IHC) was performed as previously described (*21*). Briefly, after being deparaffinized in xylene, rehydrated in descending alcohol gradients and rinsed with PBS, tissue sections were subjected to heat-mediated antigen retrieval in citrate buffer (pH 6.0) and then with 0.5% H_2_O_2_ in methanol for 10 min to remove the endogenous peroxidase followed by blocking with normal serum. The treated sections were then incubated with primary antibodies at 4°C overnight followed by incubation with a secondary antibody at room temperature for 50 min. After washing with PBS, sections were incubated with ABC reagent (Vector Laboratories, CA, USA) for 30 min, and then reacted with diaminobenzidine (DAB, Vector Laboratories, CA, USA) for 3 min. The resulting sections were then counterstained with hematoxylin until the desired stain intensity developed. Fiji ImageJ software was used to obtain data from most IHC images for quantification and statistical analyses.

Same score criteria were used across all tissue sections, including the stain intensity of target cells and the percentage of cells in a population having positive stain. The stain intensity scores were defined as 0 for unstained, 1 for light yellowish stain, 2 for claybank color stain, and 3 for brownish stain. The positive stain scores were defined as 0 for 0 ~ 5% stain positivity among cells, 1 for 6 ~ 25%, 2 for 26 ~ 50%, 3 for 51 ~ 75%, and 4 for > 75%. A comprehensive positive score (CPS) was then calculated from the staining intensity and the percentage of positive cells, which was defined as (-) for 0 or negative, (+) for weakly positive with CPS of 1 - 4, (++) for moderately positive with CPS of 5 - 8, and (+++) for strongly positive with CPS of 9 - 12. In subsequent analyses, CPS ≤ 4 was regarded as having a low expression level while CPS > 4 a high expression level.

**RNA-Seq**

Total RNA was isolated from TRCs using TRIzol reagent (Invitrogen, CA, USA) and the quantity and purity were monitored using NanoDrop ND-1000 (NanoDrop, Wilmington, DE, USA) as well as Bioanalyzer 2100 (Agilent, CA, USA). OligodT [magnetic](javascript:;) beads (25-61005, Thermo Fisher, CA, USA) enriched mRNAs were [fragmented](javascript:;). cDNAs were synthesized from the fragmented RNA using a Reverse Transcriptase (Invitrogen SuperScript™ II Reverse Transcriptase, CA, USA), and then sequenced by Illumina Novaseq™ 6000 (LC Bio Technology Co., Ltd. Hangzhou, China). The obtained RNA-Seq raw data were uploaded to the Sequence Read Archive (SRA) database of the National Center for Biotechnology Information (NCBI) (<https://www.ncbi.nlm.nih.gov/>) with the accession number PRJNA673935.

**ChIP-Seq, ChIP-qPCR and related bioinformatics**

The DMSO- or sulfarotene-treated TRCs were crosslinked in 1% formaldehyde solution at room temperature for 10 min and quenched with 125 mM glycine. After fragmentation, the chromatin segments were pre-cleared and then immunoprecipitated with 5 μL of antibodies against RARα (sc-515796, Santa Cruz) as described (4). After DNA library construction and amplification, Illumina HiSeq/NextSeq platform (Hiseq 4000, Illumina) was used for sequencing.

Target DNA abundance in ChIP immunoprecipitated and eluted DNA fragments were assessed by qPCR against the input and IgG as controls using the primers listed in Supplementary Table 3, which were designed to achieve products of 50–200 bp based on the open reading frames of the predictive RAR binding sequence of *SOS2*.

Bowtie 2 (version 2.2.1) was used to compare the filtered clean reads to human genome (ENSEMBL96, hg38) and MACS2 (version 2.1.0) was used to identify a credible sequence enrichment region, namely the peaks of the transcription factor binding region, from the short sequence alignment results. A *P-*value of 0.01 was used as the screening threshold. ChIPseeker (version 3.11) was used to annotate the difference peaks obtained in the previous step, and a 2-kb region (as counts per million) from upstream to downstream of the transcription start site (TSS) was selected as the putative promoter region. MAnorm (version 1.1.4) software was used to analyze difference samples based on ChIPseeker annotation and then peaks of significant difference were selected (|M-value| ≥ 0.5, *P-*value < 0.05). ChIP-Seq data was deposited into the Sequence Read Archive (SRA) database of National Center for Biotechnology Information (NCBI) (<https://www.ncbi.nlm.nih.gov/>) under accession number PRJNA673935. A heat map of the genes identified in ChIP-Seq analysis was generated using the Deeptools 3.0 package. The peaks in ChIP-Seq associated with binding to within 5 kb of the *SOS2* gene were visualized using IGV (version 2.5.3).

**Luciferase reporter assay**

The plasmids for RARα overexpression (pLenO-GTP-C-3XFlag-RARα), RARα control (pLenO-GTP-C-3XFlag-RARα-NC), and reporter constructs containing RARα-binding elements of human wild-type SOS2 (G0161632-1, SOS2-promotor-WT (TCATCTG, TCCCTAT)) and mutant SOS2 (G0161632-2, SOS2-promotor-MU (AGTCGAC, AGGTATA)) were constructed by Zorin Biological Corporation (Shanghai, China). The RARα gene was amplified by PCR using primer sets of forward 5’-TAGAGCTAGCGAATTCATGGCCAGCAACAGCAGCTCC-3’ and reverse 5’-CTTTGTAGTCGGATCCCGGGGAGTGGGTGGCCGG-3’. The promoter of human SOS2 gene was cloned into a dual-luciferase reporter vector pGL3 basic to generate the pGL3-SOS2-WT and pGL3-SOS2-MUT. Plasmids were transiently transfected into 293T cells. After 48 h culture, a Dual-Luciferase® Reporter Assay System was used for assessing luciferase activities according to manufacturer’s instructions (E1910, Promega, USA).

**RAS activation assay**

Levels of GTP-occupied RAS in HCC TRCs or tumor tissues were measured using a RAF pull-down assay kit (#8821, Cell Signaling Technology, USA) according to the manufacturer’s protocol. Briefly, snap-frozen tissues or TRCs were homogenized on ice in lysis buffer. Cellular debris was removed by centrifuging at 15,000 g for 20 minutes at 4°C. Protein concentrations were determined. About 1,000 μg of lysates were incubated for 50 min at 4°C with glutathione affinity agarose beads coated with GST-RAF-RBD domain, which can specifically pull-down the GTP-bound form of RAS proteins. The beads were then washed 3 times with washing buffer. Active RAS was analyzed by immunoblotting with an anti-RAS primary antibody (1:2,000, Cell Signaling Technology, USA) using GAPDH as a sample loading control. The intensities of the bands generated from western blotting were quantified using ImageJ.

**Statistical analysis**

Comparisons between 2 groups of normally distributed data were made using Student's *t*-test unless otherwise indicated. Multiple groups were compared by two-way ANOVA and Tukey's post hoc test. The Kaplan-Meier log-rank (Mantel-Cox) test based on the log-rank statistic was employed to determine the significant differences between 2 or more survival curves. Univariate and multivariate analyses were evaluated by Cox proportional hazard regression, with a HR of 95% confidence interval. GraphPad Prism was used for analyses of the overall survival and cumulative recurrence rates. Statistical analyses were also performed using SPSS version 22.0 software (IBM SPSS Statistics, Chicago, IL, US). The categorical data were subject to chi-square or Fisher’s exact test. A *P*-value < 0.05 was considered to be statistically significant. Results are expressed as the group mean ± SD of 3 independent experiments. **P* < 0.05, ***P* < 0.01, ****P* < 0.001, **** *P* < 0.000. ns, not significant.

**Supplementary Figures**


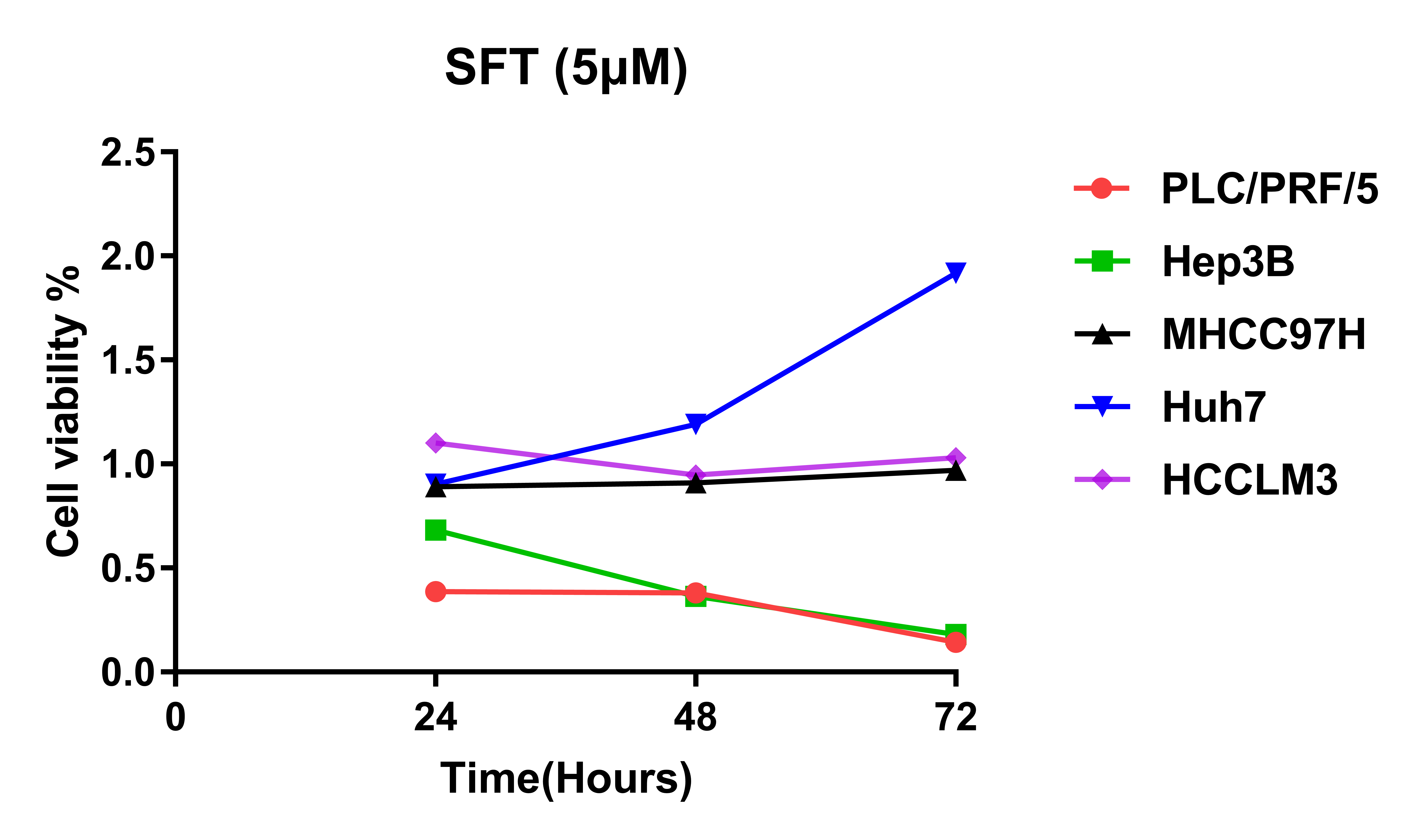


**Supplementary Fig 1. The cell viability of sulfarotene for HCC cell lines was determined in the CCK8 assay after treatment for 24h, 48 h and 72h (n = 3).**


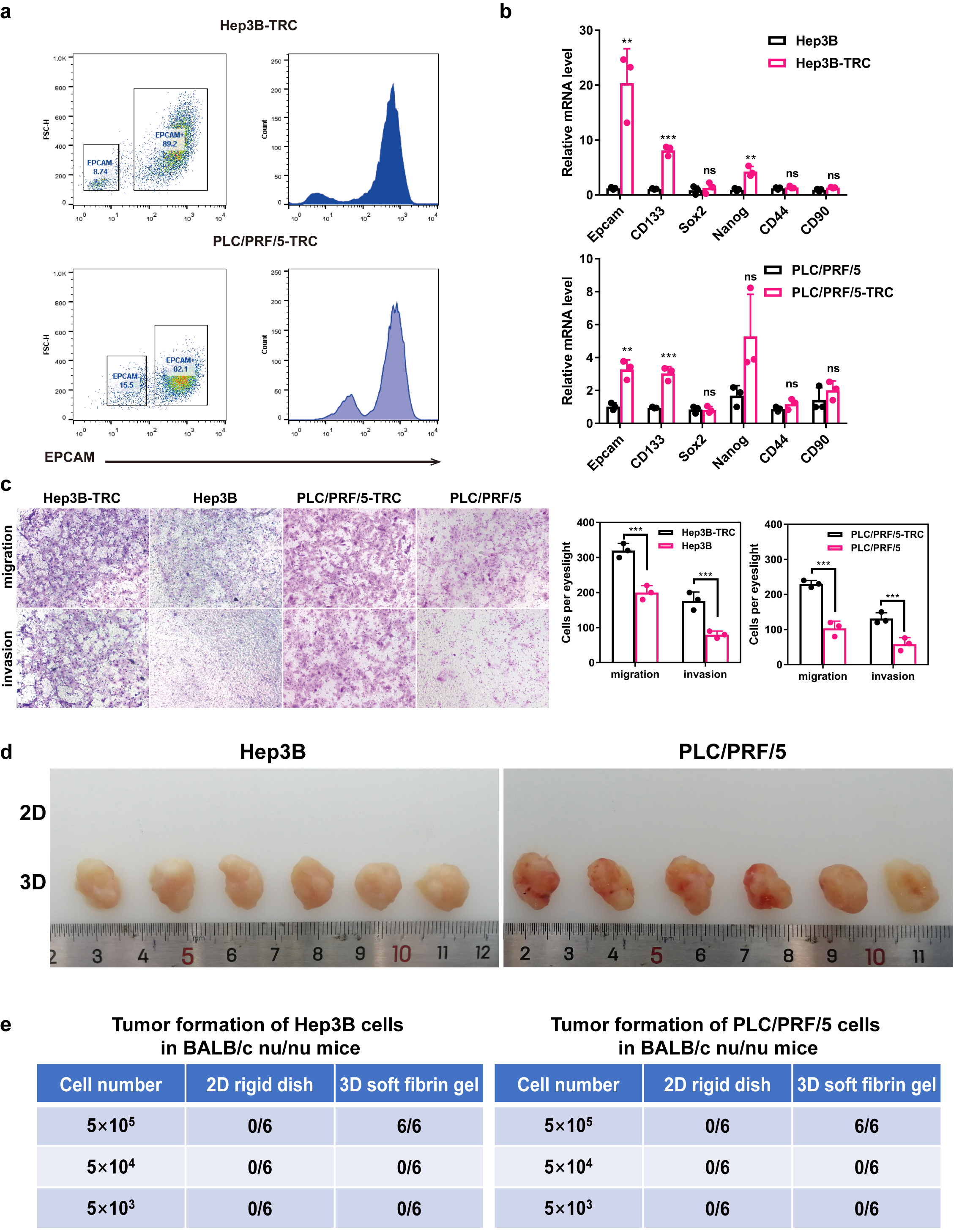


**Supplementary Fig 2. Identification and characterization of HCC cancer stem cell like TRCs.** **a** Flow cytometry analysis results of EpCAM+ populations in the HCC TRCs derived from Hep3B cells (upper panel) and PLC/PRF/5 cells (lower panel). **b** Enrichment of cancer stem cell markers in the selected HCC TRCs. Alterations in the mRNA expression levels of various cancer stem cell biomarkers as indicated in Hep3B-TRCs and PLC/PRF/5-TRCs over their respective parental cells were determined by qRT-PCR (n = 3). **c** Enhanced migration and invasion abilities of TRCs, as shown in the representative images (left panel) and quantification (right panel) of transwell migration and invasion of the selected Hep3B-TRCs and PLC/PRF/5-TRCs, compared to unselected parental Hep3B and PLC/PRF/5 cancer cells. **d** Unique tumorigenicity of the selected HCC TRCs. 5 × 10^5^ each of the Hep3B and PLC/PRF/5 cells cultured on 2D rigid dishes and the same number of the selected Hep3B-TRCs and PLC/PRF/5-TRCs in 3D soft fibrin gels were injected subcutaneously to the flanks of 6-week old BALB/c athymic nude mice. After 30 days, the volumes of the grown-up xenograft tumor nodes were photographed and measured (n = 6). **e** Tumor formation rates of different amounts of HCC cancer cells and derived TRCs showed as the number in every 6 inoculums that formed tumor nodes. Data are presented as the mean ± SD of 3 independent experiments; **P* < 0.05, ***P* < 0.01, ****P* < 0.001, Tukey's post hoc test. ns, not significant.





**Supplementary Fig 3. Comparative chemical structures of all-trans retinoic acid (ATRA), acyclic retinoid (ACR) and sulfarotene (SFT or WYC-209).**


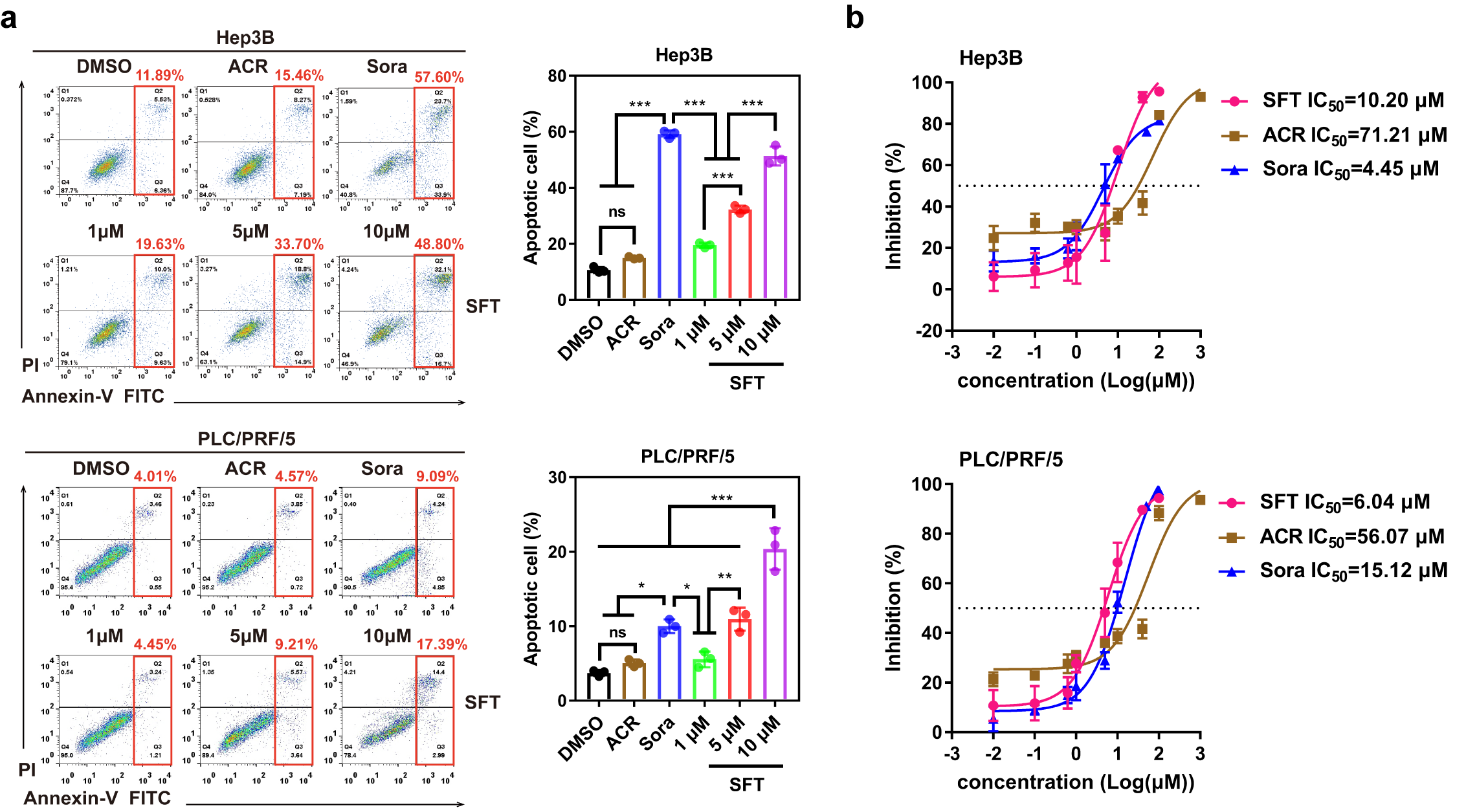


**Supplementary Fig 4. Sulfarotene inhibits proliferation and induces apoptosis of HCC cell lines. a** The apoptotic effect of sulfarotene (SFT) at 1.0, 5.0 or 10 μM on HCC cell lines Hep3B and PLC/PRF/5 was determined by flow cytometry with Annexin V and PI double staining, compared to 0.1% DMSO in the medium as carrier, 10 μM acyclic retinoid (ACR), and 10 μM sorafenib (Sora) (n = 3). **b** The IC50 values of sulfarotene, ACR and sorafenib for HCC cell lines were determined in the CCK8 assay after treatment for 48 h (n = 3). Data are presented as the mean ± SD of 3 independent experiments; **P* < 0.05, ***P* < 0.01, ****P* < 0.001, Tukey's post hoc test. ns, not significant.


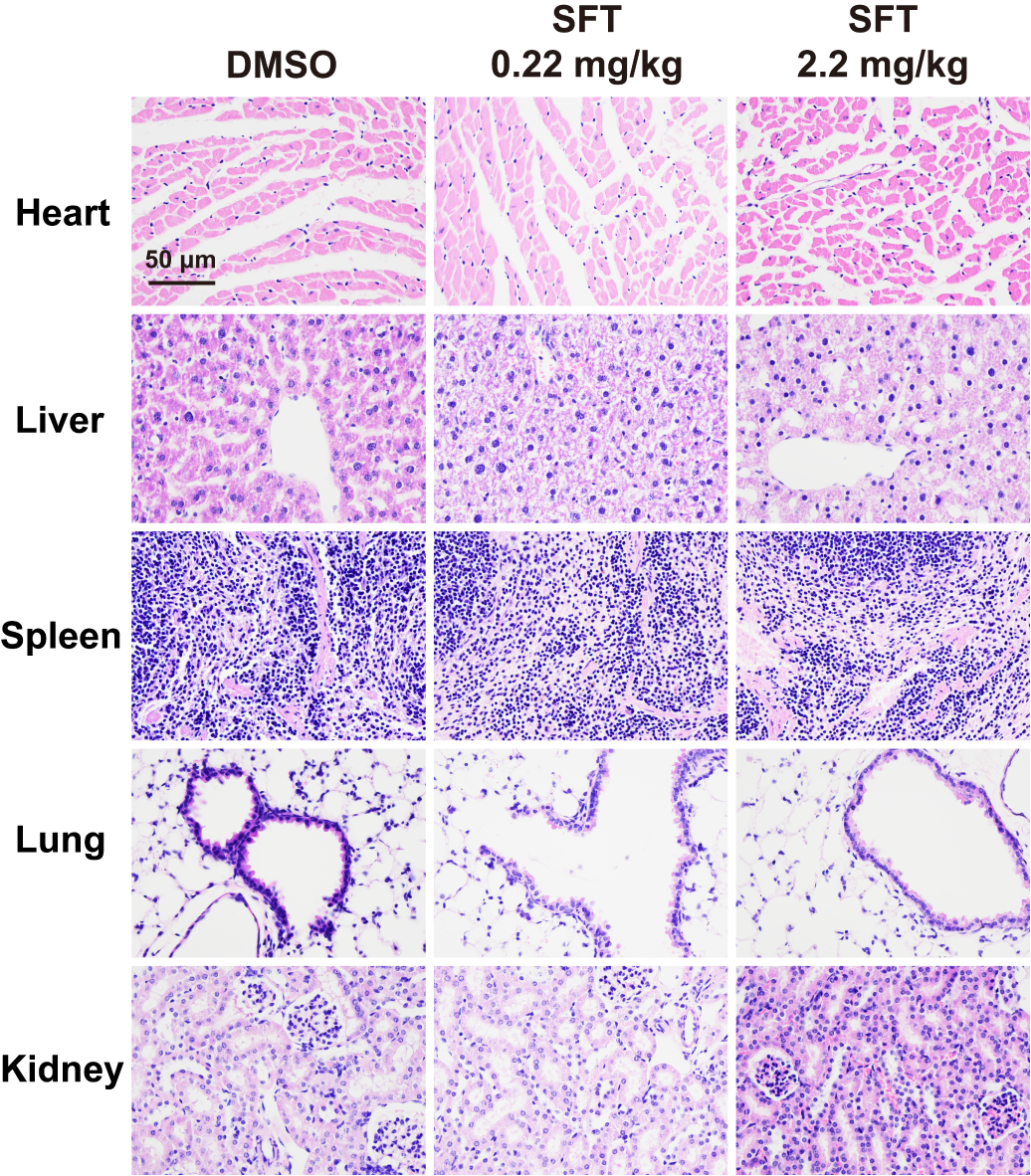


**Supplementary Fig 5. Representative hematoxylin and eosin (HE)-stained sections of mouse tissues under treatment.** Sections of the heart, liver, spleen, lung and kidney were HE stained and presented as photographs from immunodeficient BALB/c nu/nu mice hosting subcutaneously the transplanted 5 × 10^5^ TRCs in the flanks under treatment regimens with sulfarotene at 0.22 and 2.2 mg/kg.


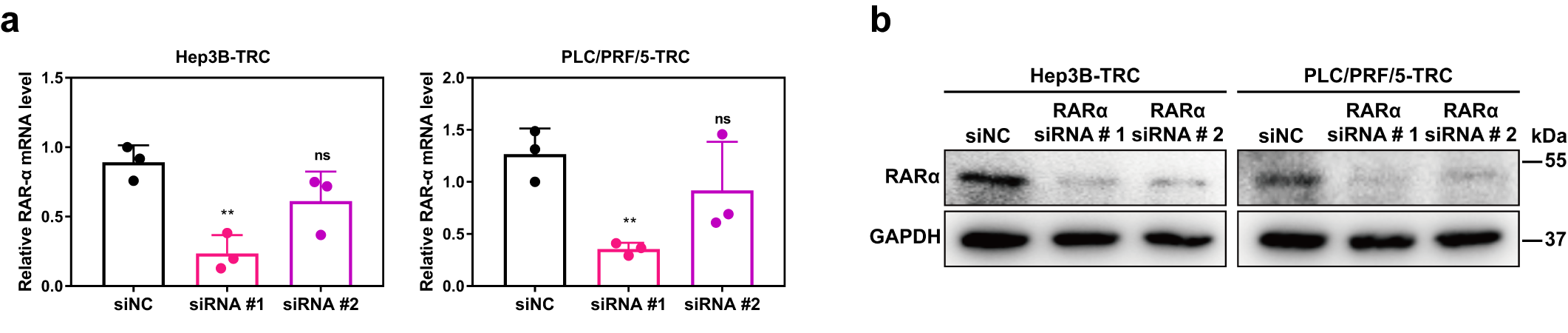


**Supplementary Fig 6. The silencing efficiency of two siRNAs targeting RAR alpha. Hep3B-TRCs and PLC/PRF/5-TRCs were transfected with siRNA#1 and siRNA#2 that specifically target RAR****α, and the mRNA (a) and protein (b) levels of RARα were measured.** **siNC, negative control siRNA.**


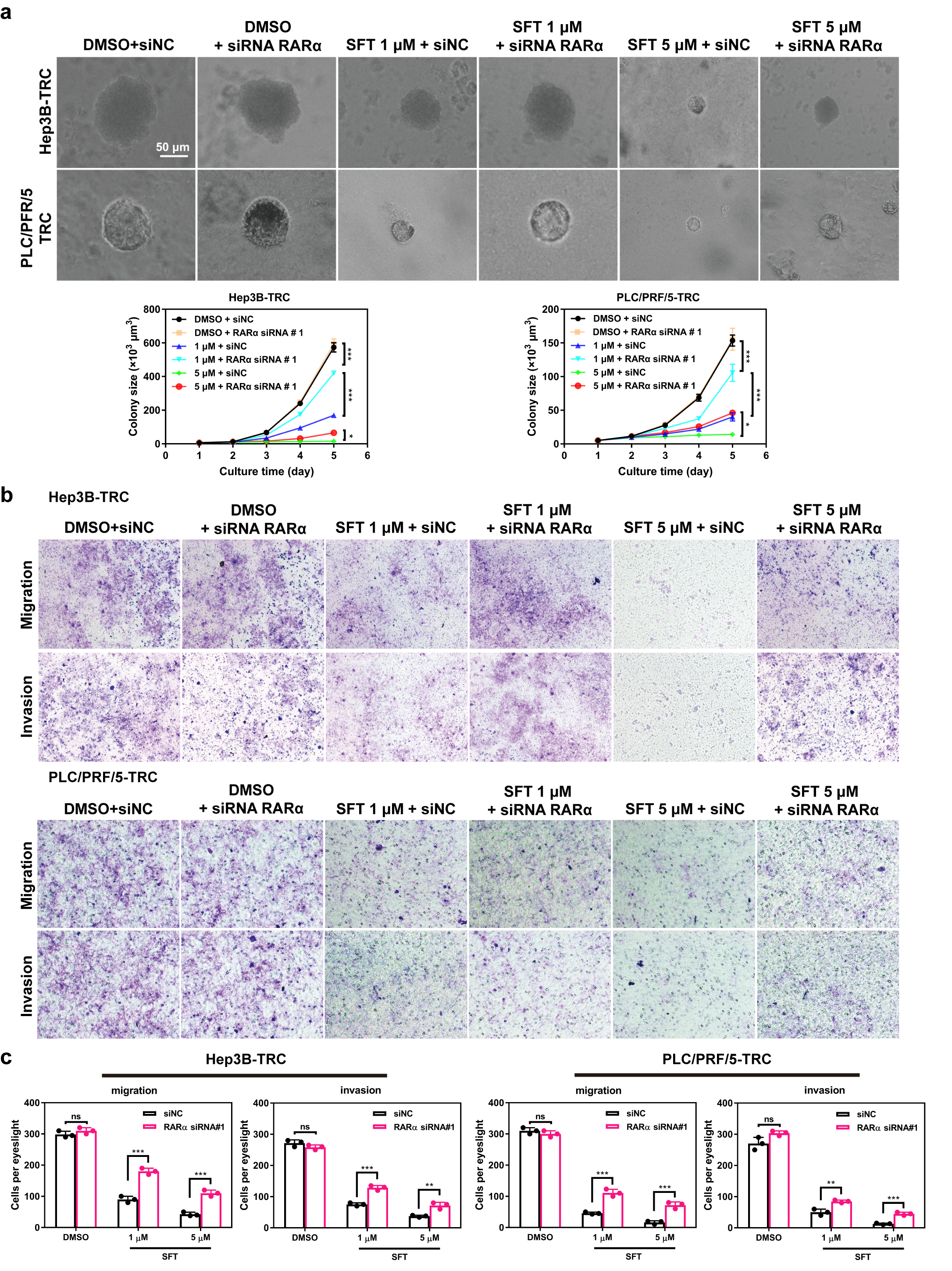


**Supplementary Fig 7. Silencing of RARα rescues the growth and metastasis of the HCC TRCs from inhibition by sulfarotene. a** Rescue of colony spheroid formation and growth derived from HCC TRCs by silencing RARα from inhibition by sulfarotene. Hep3B-TRC and PLC/PRF/5-TRC were transfected with RARα siRNA#1 and then treated with 1 or 5 μM sulfarotene compared to siNC. The changes in colony spheroid sizes = after drug treatment for 4 days were determined (n = 3). Tukey's post hoc test. **b-c** Rescue of migration and invasion of HCC TRCs by silencing RARα from inhibition by sulfarotene. Hep3B-TRC and PLC/PRF/5-TRC were transfected with RARα siRNA#1 compared to negative control siNC in 3D soft fibrin gels. After 24 h, transfected TRCs were then treated with 1 or 5 μM sulfarotene for 3 days. After treatment, TRCs that survived were pipetted gently to singlet cells, transferred to culture in the top chamber of a transwell for 2 days and processed as described in the Materials and Methods. The cells per field under microscopic examination were photographed (b) and counted (c) (n = 3). DMSO, medium with 0.1% DMSO. siNC, negative control of siRNA. SFT, sulfarotene. Data are presented as the mean ± SD of 3 independent experiments; **P* < 0.05, ***P* < 0.01, ****P* < 0.001. ns, not significant.


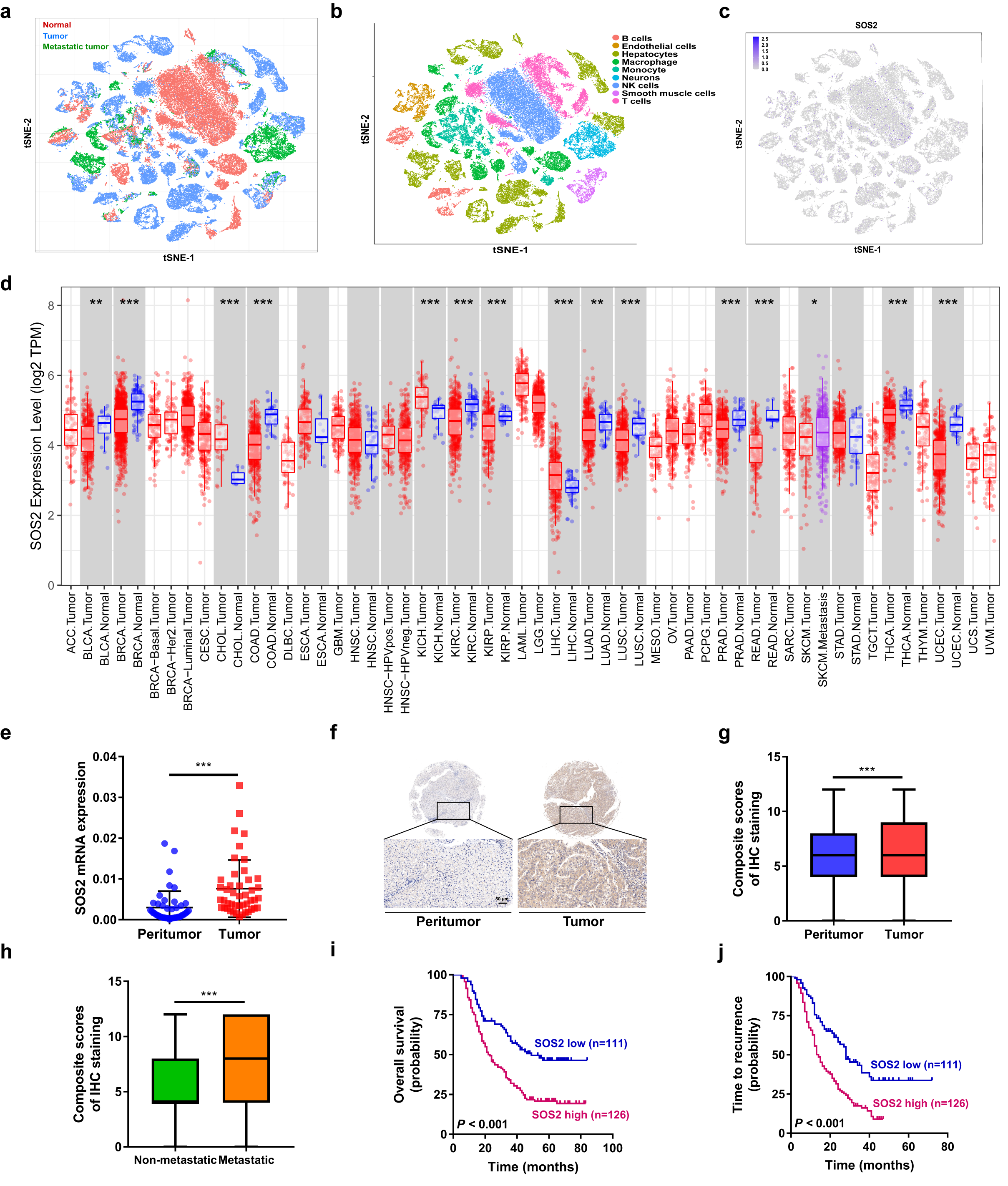


**Supplementary Fig 8. *SOS2* is an oncogenic factor in HCC TRCs. a** A tSNE plot for the differences among the metastatic tumor, primary tumor node, and the normal tissue. **b** tSNE plots for distinct cell subsets in HCC samples from patients. **c** Distribution of *SOS2* expressing cells in the tSNE plots. **d** The expression levels of *SOS2* in a number of human tumors were compared to that in the peritumoral tissues as abstracted from the TGCA database. ACC, adrenocortical carcinoma; BLCA, bladder urothelial carcinoma; BRCA, breast invasive carcinoma; CESC, cervical squamous cell carcinoma and endocervical adenocarcinoma; CHOL, cholangiocarcinoma; COAD, colon adenocarcinoma; DLBC, diffuse large B-cell lymphoma; ESCA, esophageal carcinoma; GBM, glioblastoma multiforme; HNSC, head and neck squamous cell carcinoma; KICH, Kidney chromophobe; KIRC, kidney renal clear cell carcinoma; KIRP, kidney renal papillary cell carcinoma; LAML, acute myeloid leukemia; LGG, brain lower grade glioma; LIHC, liver hepatocellular carcinoma; LUAD, lung adenocarcinoma; LUSC, lung squamous cell carcinoma; MESO, mesothelioma; OV, ovarian serous cystadenocarcinoma; PAAD, pancreatic adenocarcinoma; PCPG, pheochromocytoma and paraganglioma; PRAD, prostate adenocarcinoma; READ, rectum adenocarcinoma; SARC, sarcoma; SKCM, skin cutaneous melanoma; STAD, stomach adenocarcinoma; TGCT, testicular germ cell tumors; THCA, thyroid carcinoma; THYM, thymoma; UCEC, uterine corpus endometrial carcinoma; UCS, uterine carcinosarcoma; UVM, uveal melanoma. **e** qRT-PCR analysis revealed that *SOS2* mRNA levels in HCC tumor foci from 45 patients were significantly higher than that in peritumoral tissues compared to GAPDH as an internal reference. **f** Representative immunohistochemical (IHC) staining for SOS2 in the clinically confirmed HCC foci compared to the paired non-tumor liver tissue. IHC staining indicated that SOS2 was widely distributed in the cytoplasm of tumor cells (*inset*). **g** Scores of immunochemistry staining of SOS2 on the sections of pairs of tumor and peritumor tissues from 237 human HCC patients. The horizontal lines in the box plot (minimum to maximum) represent the median and the interquartile ranges. **h** The intratumoral SOS2 expression was significantly higher in the pulmonary metastatic foci than in non-metastatic pulmonary tissues. **i-j** Inverse association of SOS2 levels with OS and TTR in HCC patients. The OS time (i) and TTR time **(**j**)** in HCC patients were stratified by and correlated with the levels of SOS2 in 2 groups: the high SOS2 group with CPS > 4 and low SOS2 group with CPS ≤ 4. The Kaplan–Meier curves of OS and TTR of 237 HCC patients were plotted. The *P*-values were determined by the log-rank test. Data are presented as the mean ± SD; ****P* < 0.001.


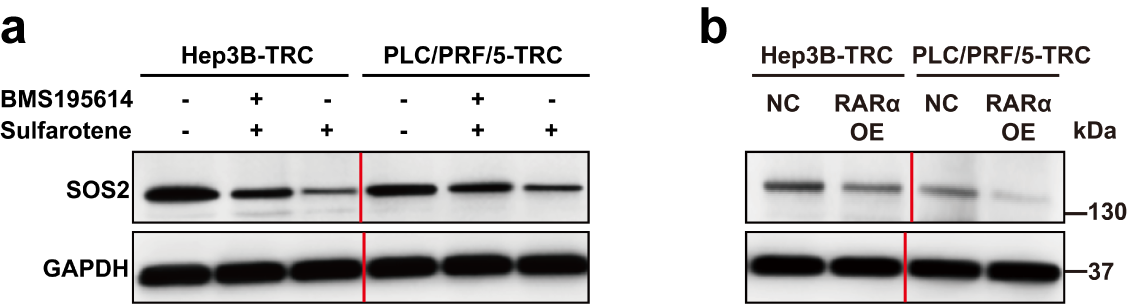


**Supplementary Fig 9. Sulfarotene via RARα downregulates the expression of SOS2. a** 5.0 μM sulfarotene significantly reduced SOS2 protein levels while 1.0 μM of the RARα antagonist BMS195614 significantly rescued the reduction in Hep3B-TRCs and PLC/PRF/5-TRCs cultured in 90 Pa fibrin gels for 24 h. **b** Stable overexpression of RARα significantly repressed the levels of SOS2 in TRCs after cultured for 5 days.


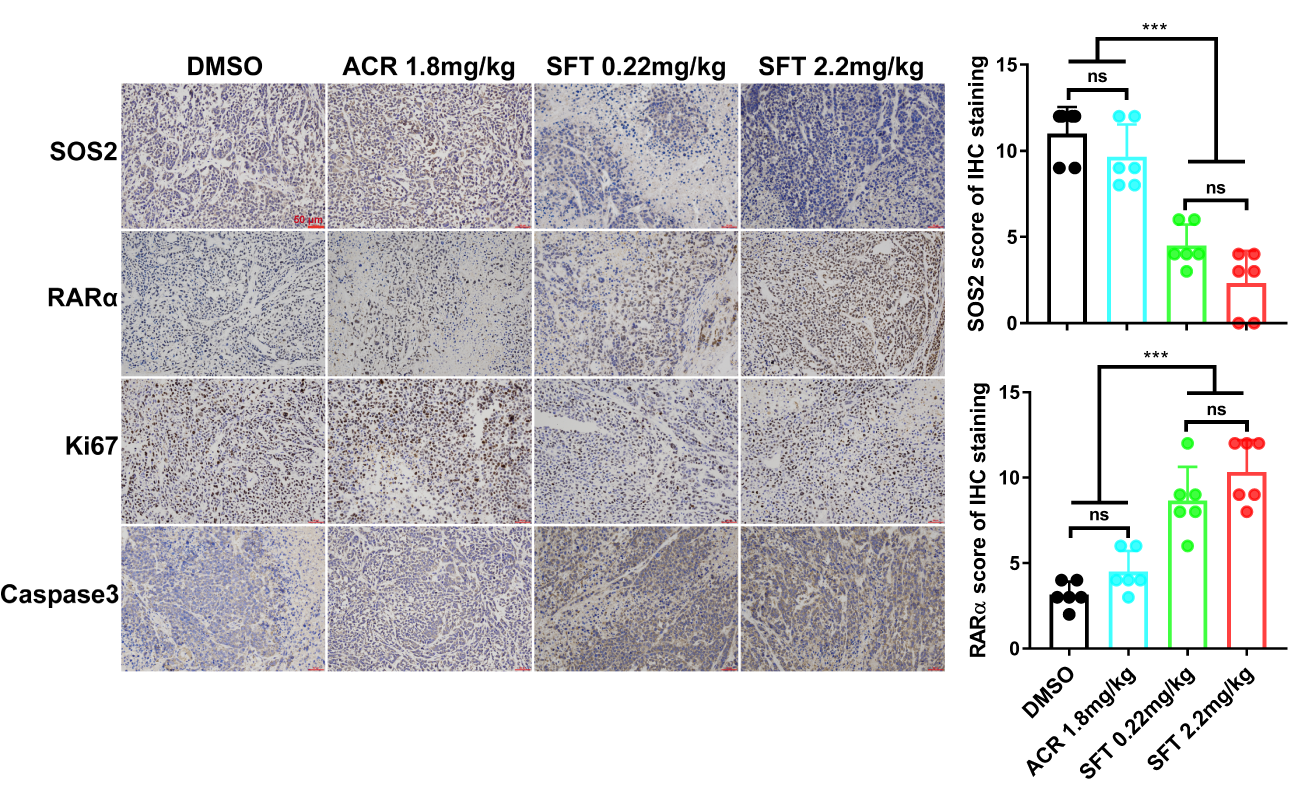


**Supplementary Fig 10. Sulfarotene upregulates RARα while downregulating SOS2 in tumors derived from HCC TRCs.** Representative IHC images (left) of sections of xenograft tumors from nude mice subcutaneously injected with Hep3B-TRCs and treated with 0.22 and 2.2 mg/kg sulfarotene compared to 1.8 mg/kg ACR and DMSO for the levels of SOS2, RARα, Ki-67 and Caspase-3. Bar graphs (right) show the IHC score ± SD in each group. Tukey's post hoc test. ****P* < 0.001. ns, not significant.


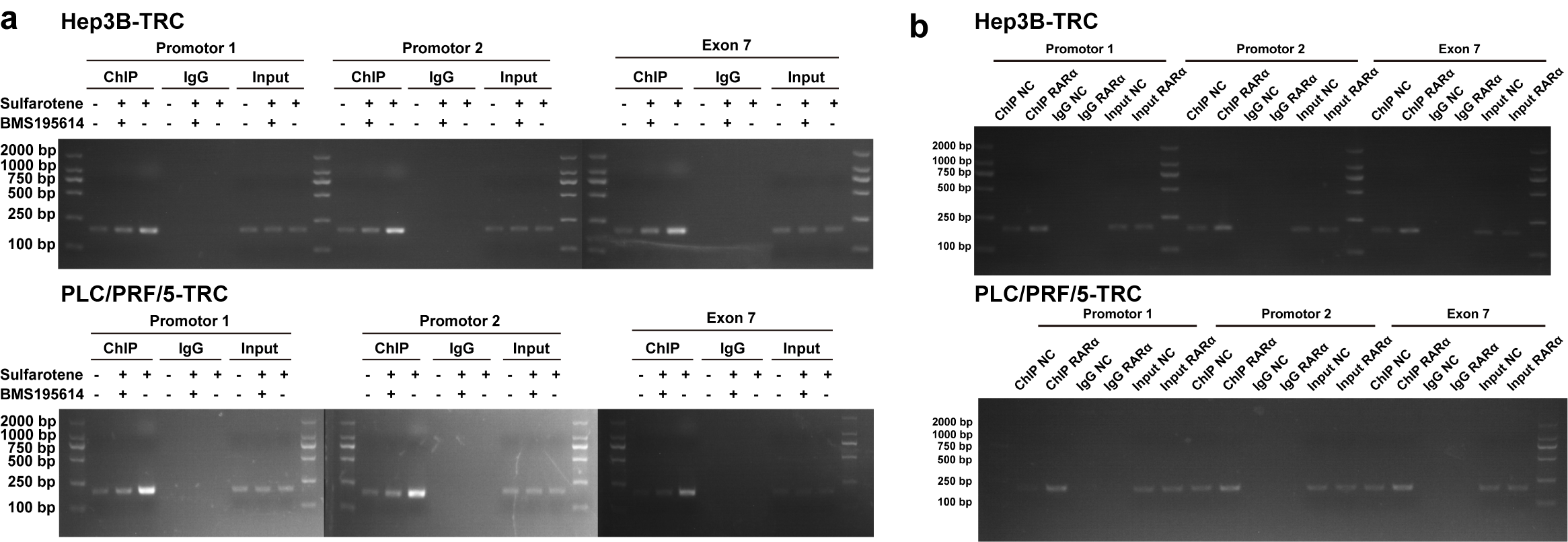


**Supplementary Fig 11. Transcriptional regulation of *SOS2* by RARα that is targeted directly by sulfarotene.** **a-b** Agarose gel electrophoresis revelation of ChIP-PCR signals amplified from RARα-binding regions in the *SOS2* gene locus in HCC TRCs, which was either stably overexpressing RARα or treated with 5 μM sulfarotene and/or BMS195614 for 5 days as indicated.


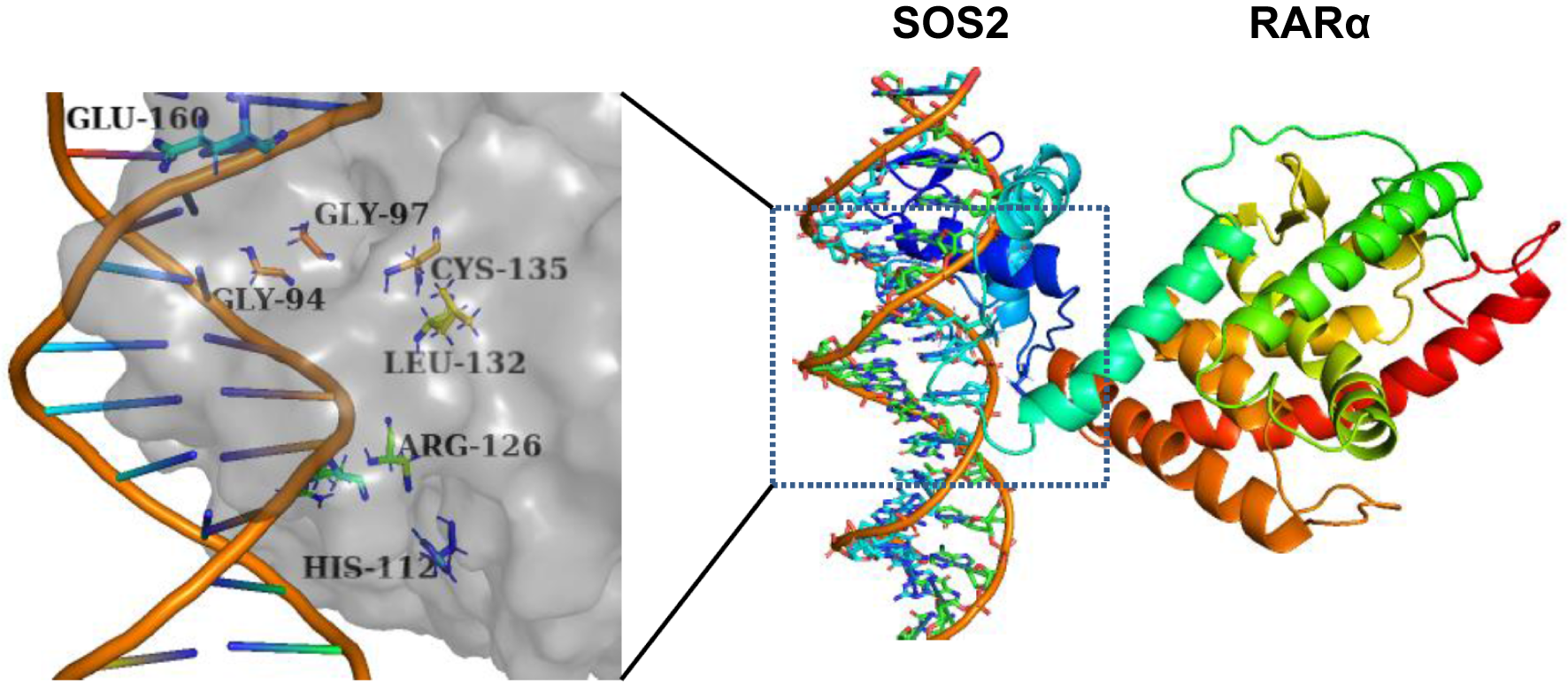


**Supplementary Fig 12. Structure docking simulation shows the specific docking interactions between the RARα transcription factor and the DNA double helix of the putative RARα-binding element in exon 7 of *SOS2*.**


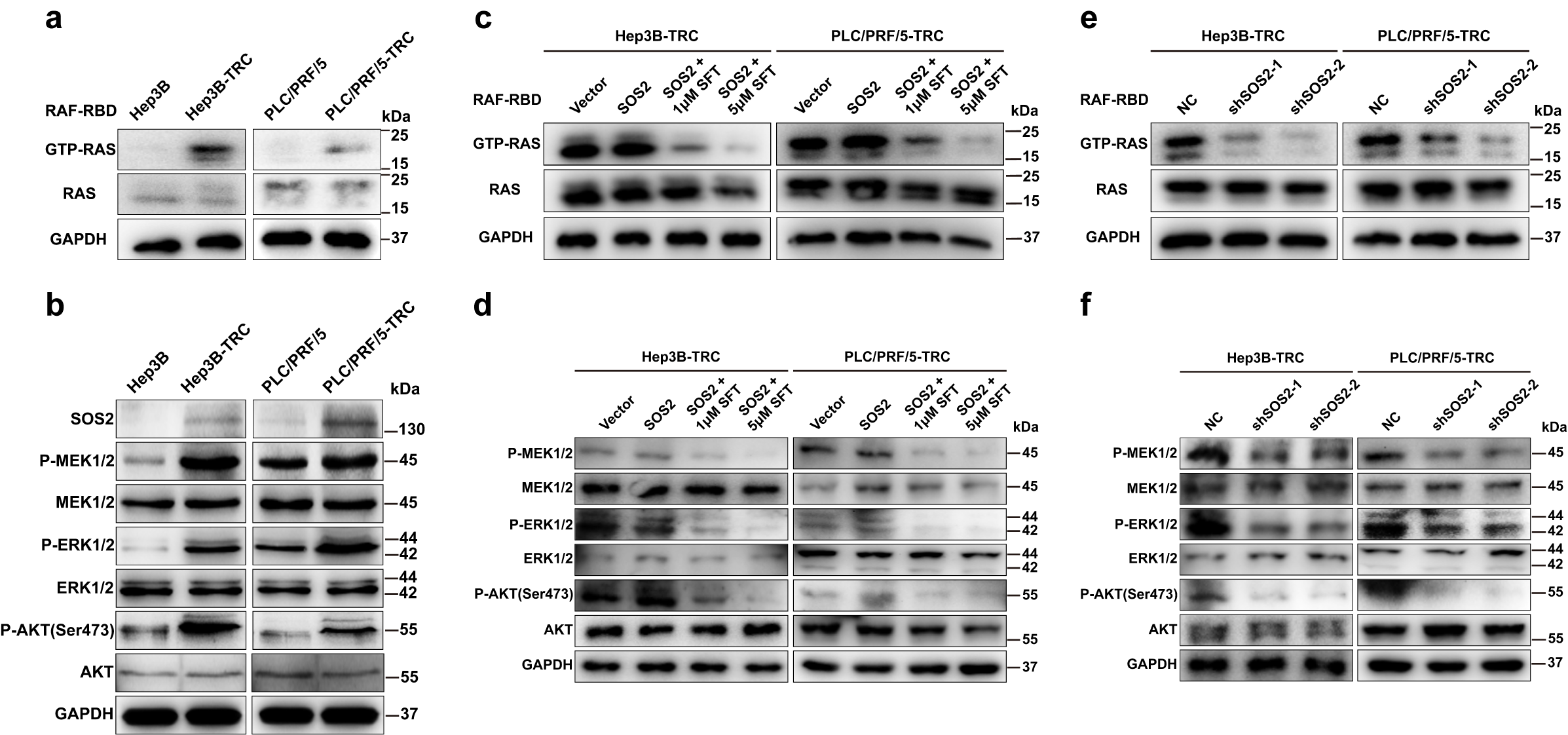


**Supplementary Fig 13. Sulfarotene inhibits *SOS2*-RAS nexus and associated signaling pathways in HCC TRCs. a** Significant activation of RAS-GTPase in HCC TRCs. Blots of RAF-RBD precipitated RAS from whole cell lysates of Hep3B-TRCs and PLC/PRF5-TRCs compared to Hep3B and PLC/PRF5 cell lines illustrated significant increases in GTP-bound RAS in TRCs. **b** Increases in p-MEK1/2, p-ERK1/2 and p-AKT (Ser473) levels in Hep3B-TRCs and PLC/PRF5-TRCs coincided with increases of SOS2 and GTP-RAS (a). **c-d** Effects of overexpression of SOS2 on GTP-RAS and associated downstream pathways in response to sulfarotene treatment. Hep3B-TRCs and PLC/PRF/5-TRCs that stably overexpressed SOS2 were treated with sulfarotene at concentrations of 1.0 and 5.0 μM for 48 h, and the levels of active RAS, p-MEK1/2, p-ERK1/2, and p-AKT were analyzed by western blotting. **e-f** Significant inhibition of RAS activation and associated PI3K/AKT and MAPK pathways by direct SOS2 knockdown. Hep3B-TRCs and PLC/PRF/5-TRCs were subjected to infection with viral particles carrying shRNAs that targeted *SOS2*. Three days later, the levels of active RAS, p-MEK1/2, p-ERK1/2, and p-AKT were analyzed by western blotting. GAPDH was used as the loading control.


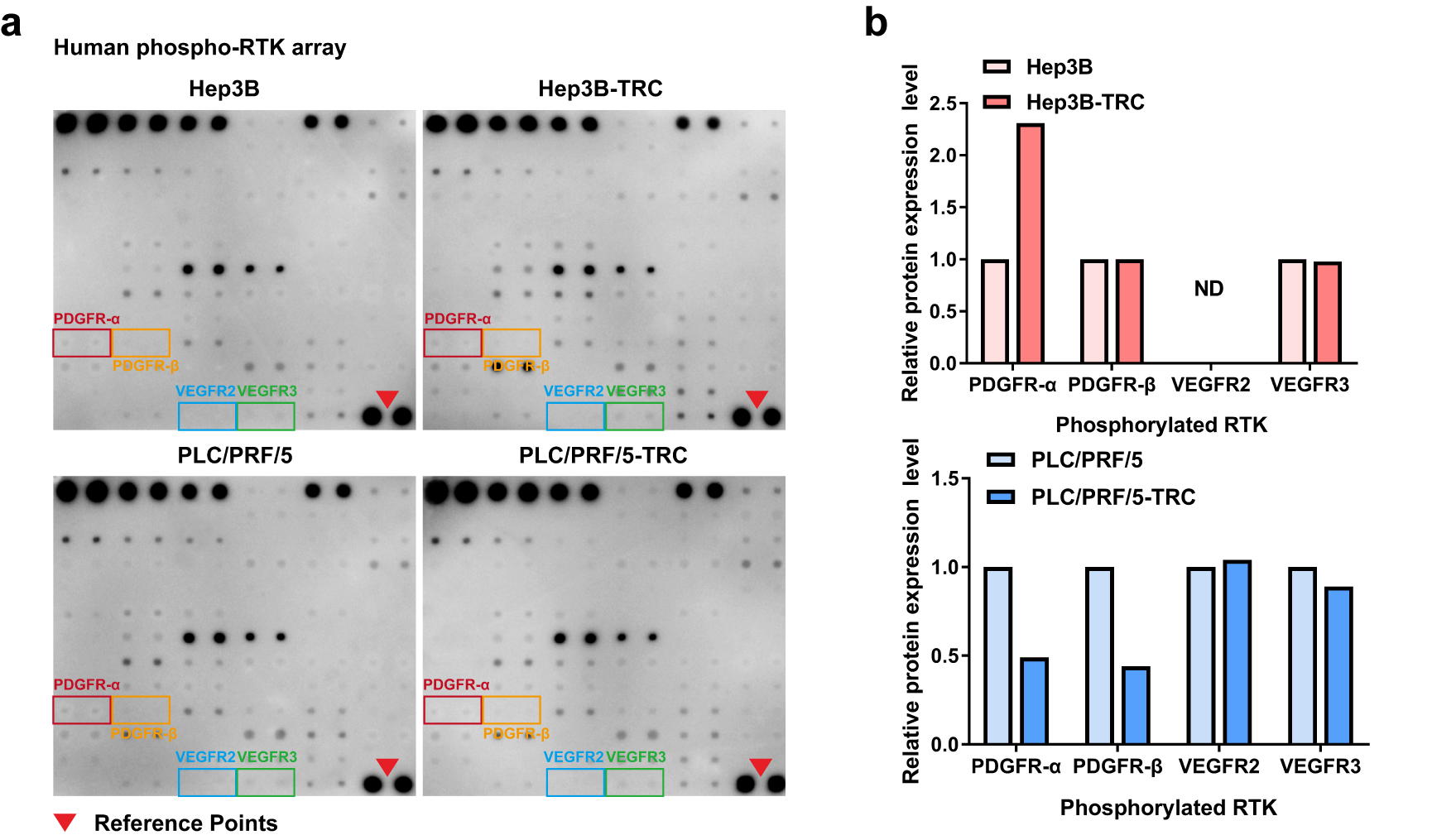


**Supplementary Fig 14. Changes in the phosphorylation (active) levels of human RTKs in the HCC TRCs relative to the parental cancer cells**. Several known targets of sorafenib such as PDGFR-α, β and VEGFR3 were found to be downregulated in PLC/PRF/5-TRCs (Right). Array blot images (Left) were quantified by densitometry using ImageJ software. ND, not detected.


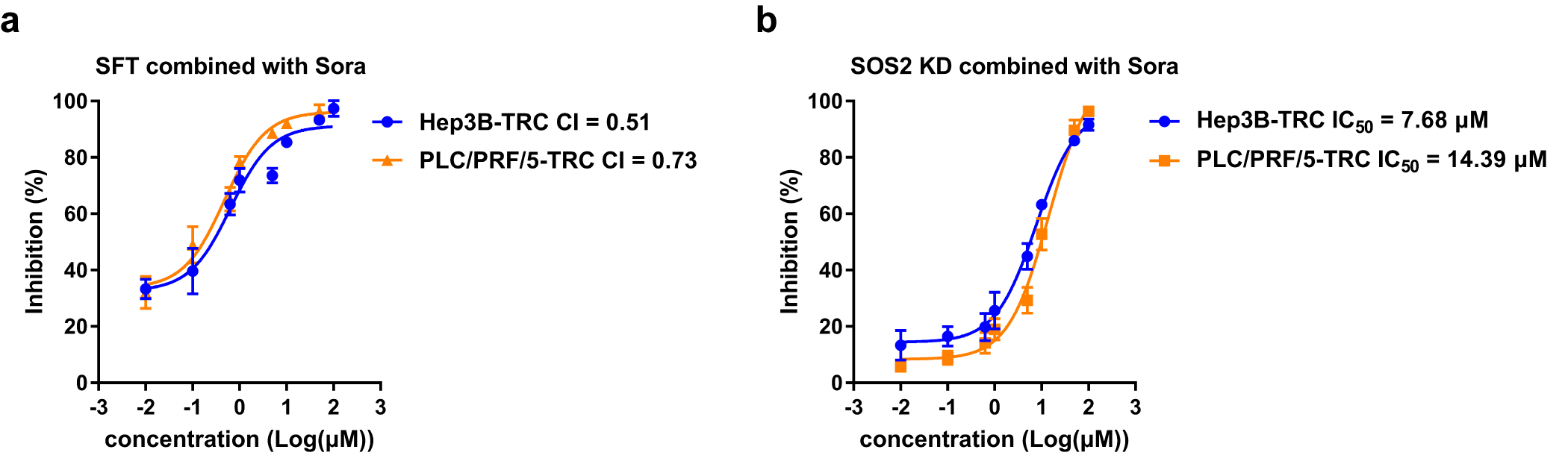


**Supplementary Fig 15. The effect on the combination between sulfarotene and sorafenib. a** The combination index (CI) of sulfarotene combined with sorafenib for HCC TRCs were determined in the CCK8 assay after treatment for 48 h (n = 3). **b** The IC50 values of SOS2 knockdown combined with sorafenib for HCC TRCs were determined in the CCK8 assay after treatment for 48 h (n = 3).


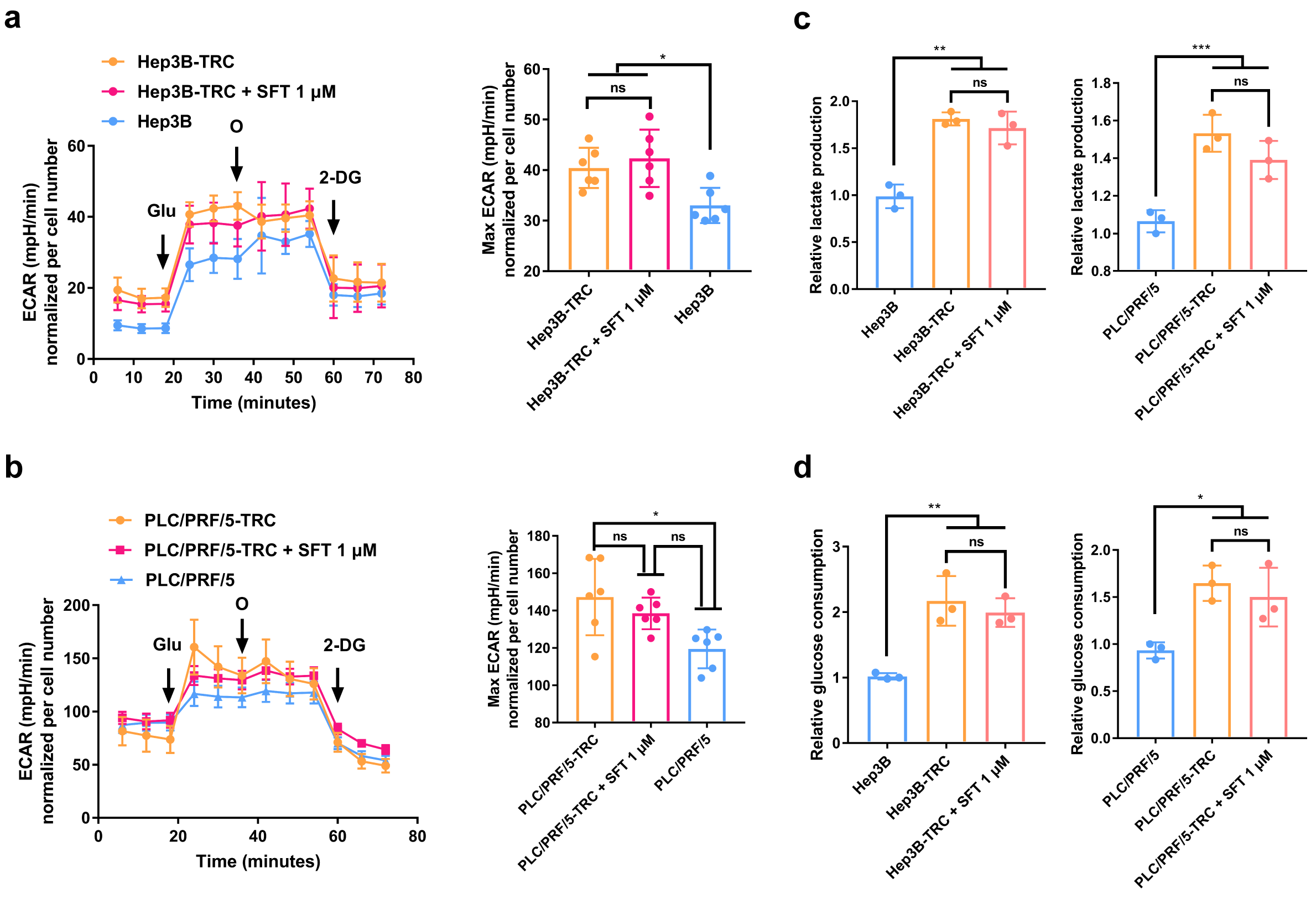


**Supplementary Fig 16.** **SFT do not modulate aerobic glycolysis in HCC TRCs.** **a-b** Effects on extracellular acid ratio (ECAR) among HCC cell lines, HCC TRCs and HCC TRCs treated with SFT. **c-d** Lactate production (b) and glucose consumption (c) in HCC TRCs and HCC TRCs treated with SFT compared to relative control cells.

**Table S1. Univariate and multivariate analyses of prognostic factors associated with OS and TTR in HCC patients (n = 237)**

| Variables | OS | | | | TTR | | | |
| --- | --- | --- | --- | --- | --- | --- | --- | --- |
|  | Univariate | Multivariate | | | Univariate | Multivariate | | |
|  | *P*-value | HR | 95% CI | *P*-value | *P** | HR | 95% CI | *P*-value |
| Age, years (> 50 *vs* ≤ 50) | 0.855 |  |  | NA | 0.729 |  |  | NA |
| Gender (female *vs* male) | 0.938 |  |  | NA | 0.402 |  |  | NA |
| HBsAg (positive *vs* negative) | 0.404 |  |  | NA | 0.699 |  |  | NA |
| Liver cirrhosis (yes *vs* no) | 0.942 |  |  | NA | 0.608 |  |  | NA |
| Serum AFP, ng/ml (≤ 20 *vs* > 20) | 0.125 |  |  | NA | **0.009** | 0.632 | 0.418-0.956 | **0.030** |
| Serum ALT, U/L (> 75 *vs* ≤75) | 0.436 |  |  | NA | 0.566 |  |  | NA |
| Tumor size (cm) (> 5 *vs* ≤ 5) | **0.000** | 2.238 | 1.493-3.356 | **0.000** | **0.000** | 2.757 | 1.784-4.260 | **0.000** |
| Tumor encapsulation (none *vs* complete) | **0.000** | 3.473 | 2.199-5.486 | **0.000** | **0.000** | 3.053 | 1.921-4.853 | **0.000** |
| Tumor multiplicity (multiple *vs* single) | **0.000** |  |  | NS | **0.000** |  |  | NS |
| Microvascular invasion (yes *vs* no) | **0.000** | 1.664 | 1.065-2.600 | **0.025** | **0.000** |  |  | NS |
| BCLC stage (B/C *vs* A) | **0.000** | 2.657 | 1.617-4.366 | **0.000** | **0.000** | 2.194 | 1.310-3.676 | **0.003** |
| Tumor SOS2 level (high *vs* low) | **0.000** | 1.442 | 1.012-2.054 | **0.043** | **0.000** | 1.485 | 1.040-2.120 | **0.029** |

**Abbreviations:** HCC, hepatocellular carcinoma; OS, overall survival; TTR, time to recurrence; HBsAg, hepatitis B surface antigen; AFP, α-fetoprotein; ALT, alanine transaminase; HR, hazard ratio; 95% CI, 95% confidential interval. NA, not applicable; NS, not significant. A *P* value* < 0.05 was considered to be statistically significant.

**Table S2. Clinicopathological characteristics of the 237 enrolled HCC patients**

| Characteristics | Non-pulmonary  metastasis group  (n = 110) | | Pulmonary  metastasis group  (n = 127) | | P-value |
| --- | --- | --- | --- | --- | --- |
|  | n | % | n | % |  |
| Age, years |  |  |  |  |  |
| ≤ 50 | 41 | 37.3 | 51 | 40.2 | 0.649 |
| > 50 | 69 | 62.7 | 76 | 59.8 |  |
| Gender |  |  |  |  |  |
| Female | 20 | 18.2 | 22 | 17.3 | 0.863 |
| Male | 90 | 81.8 | 105 | 82.7 |  |
| HBsAg |  |  |  |  |  |
| Negative | 17 | 15.5 | 18 | 14.2 | 0.782 |
| Positive | 93 | 84.5 | 109 | 85.8 |  |
| Cirrhosis |  |  |  |  |  |
| No | 33 | 30.0 | 31 | 24.4 | 0.334 |
| Yes | 77 | 70.0 | 96 | 75.6 |  |
| AFP (ng/mL) |  |  |  |  |  |
| ≤ 20 | 13 | 11.8 | 25 | 19.7 | 0.100 |
| > 20 | 97 | 88.2 | 102 | 80.3 |  |
| ALT (U/L) |  |  |  |  |  |
| ≤ 75 | 79 | 71.8 | 98 | 77.2 | 0.345 |
| > 75 | 31 | 28.2 | 29 | 22.8 |  |
| Tumor size (cm) |  |  |  |  |  |
| ≤ 5 | 65 | 59.1 | 52 | 40.9 | 0.005 |
| > 5 | 45 | 40.9 | 75 | 59.1 |  |
| Tumor encapsulation |  |  |  |  |  |
| None | 105 | 95.5 | 0 | 0.0 | 0.000 |
| Complete | 5 | 4.5 | 127 | 100.0 |  |
| Tumor multiplicity |  |  |  |  |  |
| Single | 47 | 42.7 | 3 | 2.4 | 0.000 |
| Multiple | 63 | 57.3 | 124 | 97.6 |  |
| Microvascular invasion |  |  |  |  |  |
| No | 80 | 72.7 | 20 | 15.7 | 0.000 |
| Yes | 30 | 27.3 | 107 | 84.3 |  |
| BCLC stage |  |  |  |  |  |
| A | 83 | 75.5 | 24 | 18.9 | 0.000 |
| B+C | 27 | 24.5 | 103 | 81.1 |  |
| SOS2 in tumor tissue |  |  |  |  |  |
| Negative | 63 | 57.3 | 34 | 26.8 | 0.000 |
| Positive | 47 | 42.7 | 93 | 73.2 |  |

**Abbreviations:** HCC, hepatocellular carcinoma; HBsAg, hepatitis B surface antigen; AFP, α-fetoprotein; ALT, alanine transaminase; BCLC, Barcelona Clinic Liver Cancer. *P* < 0.05 was considered to be statistically significant, Pearson χ2 test.

**Table S3. CHIP-qPCR primers used in the study**

| **Gene locus** | **Primer** | **Sequence (5’-3’)** | **Target Size** |
| --- | --- | --- | --- |
| SOS2 (amplicon 1) Promoter 1 | Sense primer | CCGTGCAAGTGAAGGACCT | 245 bp |
|  | Anti-sense primer | GGCTGGACAGGATATTTAACGTA |  |
| SOS2 (amplicon 2) Promoter 2 | Sense primer | CCACACCATCACCCTAAGAAAT | 223 bp |
|  | Anti-sense primer | GGACTCGGTGCCACTCTTGTAG |  |
| SOS2 (amplicon 3) Exon 7 | Sense primer | TTTGAAGATTTGGCAGAAGAGC | 139 bp |
|  | Anti-sense primer | GATACACTGGCACCAGCATAAGA |  |

**Reference**

1. Liu J, Tan Y, Zhang H, Zhang Y, Xu P, Chen J, et al. Soft fibrin gels promote selection and growth of tumorigenic cells. Nat Mater. 2012;11(8):734-41.

2. Huang W, Hu H, Zhang Q, Wu X, Wei F, Yang F, et al. Regulatory networks in mechanotransduction reveal key genes in promoting cancer cell stemness and proliferation. Oncogene. 2019;38(42):6818-34.

3. Chen J, Cao X, An Q, Zhang Y, Li K, Yao W, et al. Inhibition of cancer stem cell like cells by a synthetic retinoid. Nat Commun. 2018;9(1):1406.

4. Lee TI, Johnstone SE, Young RA. Chromatin immunoprecipitation and microarray-based analysis of protein location. Nat Protoc. 2006;1(2):729-48.
